# The hyper-activation of transcriptional enhancers in breast cancer

**DOI:** 10.1186/s13148-019-0645-x

**Published:** 2019-03-12

**Authors:** Qing-Lan Li, Dan-Ya Wang, Lin-Gao Ju, Jie Yao, Chuan Gao, Pin-Ji Lei, Lian-Yun Li, Xiao-Lu Zhao, Min Wu

**Affiliations:** 1 Hubei Key Laboratory of Cell Homeostasis, College of Life Sciences, Wuhan University, Wuhan, 430072 Hubei China; 2 Hubei Key Laboratory of Developmentally Originated Disease, Hubei Key Laboratory of Enteropathy, Wuhan University, Wuhan, 430072 Hubei China; 3 Department of Urology, Zhongnan Hospital, Wuhan University, Wuhan, 430072 Hubei China

**Keywords:** Epigenomics, Super-enhancer, H3K27ac, H4K8ac, H3K4me3, Breast cancer

## Abstract

**Background:**

Activation of transcription enhancers, especially super-enhancers, is one of the critical epigenetic features of tumorigenesis. However, very few studies have systematically identified the enhancers specific in cancer tissues.

**Methods:**

Here, we studied the change of histone modifications in MMTV-PyVT breast cancer model, combining mass spectrometry-based proteomics and ChIP-seq-based epigenomics approaches. Some of the proteomic results were confirmed with western blotting and IHC staining. An inhibitor of H3K27ac was applied to study its effect on cancer development.

**Results:**

H3K27ac and H4K8ac are elevated in cancer, which was confirmed in patient tissue chips. ChIP-seq revealed that H4K8ac is co-localized with H3K27ac on chromatin, especially on distal enhancers. Epigenomic studies further identified a subgroup of super-enhancers marked by H3K4me3 peaks in the intergenic regions. The H3K4me3-enriched regions enhancers are associated with higher level of H3K27ac and H4K8ac compared with the average level of conventional super-enhancers and are associated with higher transcription level of their adjacent genes. We identified 148 H3K4me3-enriched super-enhancers with higher gene expression in tumor, which may be critical for breast cancer. One inhibitor for p300 and H3K27ac, C646, repressed tumor formation probably through inhibiting *Vegfa* and other genes.

**Conclusions:**

Taken together, our work identifies novel regulators and provides important resource to the genome-wide enhancer studies in breast cancer and raises the possibility of cancer treatment through modulating enhancer activity.

**Electronic supplementary material:**

The online version of this article (10.1186/s13148-019-0645-x) contains supplementary material, which is available to authorized users.

## Background

The recent advances in epigenetics and genomics have revealed the critical roles of epigenetic factors in cancer. The dynamic regulation of histone acetylation and methylation becomes critical for chromatin stability, tumorigenesis, and metastasis [1–3]. Inhibitors for histone deacetylases (HDACs) have been approved to be used in clinical treatment, such as SAHA and VPA [4]. Recently, inhibitors for histone acetylation readers have become another new family of promising drugs in cancer treatment [5]. Besides these, quite a few of histone methyltransferases and demethylases have been characterized as key molecules in cancer. SET domain containing 2 (SETD2), a major H3K36me3 methyltransferase in mammalian cells, is frequently mutated in clear cell renal carcinoma, leukemia, glioma, and other cancers, and H3K36me3 catalyzed by SETD2 is involved in mRNA alternative splicing, genome stability, and DNA repair process [6–8]. Myeloid/lymphoid or mixed-lineage leukemia 1–4 (MLL1–4), the methyltransferases for H3K4, are often mutated in multiple types of cancers [1, 9]. On the other hand, histone demethylases are often related with tumorigenesis. Clinical studies found that the demethylases of H3K9me2 and me3, such as lysine (K)-specific demethylase 3A (KDM3A, also known as JMJD1A or JHDM2A) and lysine (K)-specific demethylase 4A/B/C (KDM4A/B/C), are highly expressed in cancer tissues and regulate tumorigenesis [10–12]. Thus, to understand the spatiotemporal regulation of epigenetic dynamics is critical for current cancer research.

Along with the development of next-generation sequencing (NGS), epigenomics has gained the focus of cancer research field. Especially, the reprogramming of distal enhancers has emerged as one of the important features for tumor [1, 13]. Classically, the enhancers were thought to locate within several kb from transcription start sites (TSS). However, recent studies revealed that in fact most of the enhancers are much farther away from TSS, often over several hundreds of kb away, so-called distal enhancers [13–15]. Enhancers are usually marked with H3K4me1, which is catalyzed by histone methyltransferase MLL3/4 [16, 17]. The active enhancers are further labeled with H3K27ac and bound by mediator complex, which facilitates enhancer-promoter crosstalk [13, 18, 19]. Nowadays, H3K27ac is often used as a mark to identify active enhancers and p300 is the corresponding histone acetyltransferase [20, 21]. A portion of enhancers gather together, form wide regions on chromatin with high level of H3K27ac, and extend about 10 kb and sometimes even longer, which are called super-enhancers [5, 22–24]. Super-enhancers are believed to contain high transcription activity, control the expression of master genes in the cell, and can be used for cell identification. In the tumor cells, super-enhancers are the potential critical regulatory elements on chromatin, often associated with activated oncogenes [23]. Therefore, the genomic landscapes of super-enhancers in different cancers have to be illustrated, and the molecular mechanisms regulating the activity of super-enhancers need to be elucidated.

In the current study, we utilized a mass spectrometry-based proteomic approach and studied the difference of histone modifications between tumor and normal tissues of the MMTV-PyVT breast cancer mouse model. We found many histone modifications, including H3K27ac and H4K8ac, are changed in breast cancer tissues. We further studied their genome distributions through the ChIP-seq-based epigenomic approach and identified a lot of cancer-specific super-enhancers. Further analysis established a subgroup of super-enhancers with H3K4me3 as potential important regulatory elements for cancer. Unfortunately, we did not find a chemical inhibitor for H3K4me3, but we successfully repressed tumor growth through inhibiting H3K27ac with a specific small molecule.

## Results

### The proteomic studies of the normal and breast cancer tissues

**Fig. 1:**
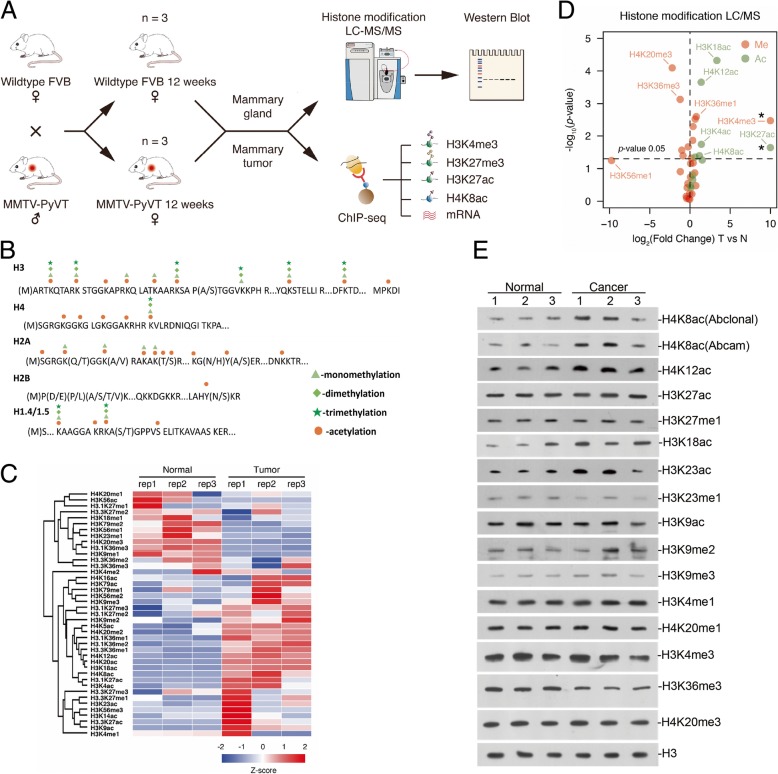
Several histone modifications altered in breast cancer development. **a** Workflow for the whole project, including mice mating, LC-MS/MS screening, Western blot validation, and ChIP-seq analysis. **b** Overview of quantified histone PTMs in core histone proteins in breast tissues. **c** Heat map of quantified histone marks found to be regulated in tumor tissue as compared to normal tissue. **d** Volcano plots representing the fold change (*x*-axis) and the significance (*y*-axis) for single histone marks. The statistical difference is calculated using the *t* test. The two histone marks H3K4me3 and H3K27ac marked with asterisk (*) were only detected in tumor by MS analysis. **e** Western blot analysis with the indicated histone marks antibodies. Whole tissue lysates were prepared from normal tissue or tumor 3 replicates

A mass spectrometry-based proteomic approach was utilized in this study to characterize histone post-translational modifications (PTMs) in the normal and breast cancer tissues of the MMTV-PyVT mice model. The experimental workflow is illustrated in Fig. 1a. Three biological replicates were included for both normal and breast cancer tissues. Histone proteins were extracted from breast tissues by acid extraction. Histones were chemically propionylated at lysine residues and peptide N-terminus using propionic anhydride and then digested with trypsin. The resulting Arg-C like histone peptides were composed of 7 to 20 amino acids and subjected to LC-MS/MS analysis operated in data-independent acquisition (DIA) mode. The dynamic histone PTMs were identified and quantified by using EpiProfile [25, 26]. Two examples for histone H3 peptide KSAPATGGVKKPHR (aa 27-40) and H4 peptide GKGGKGLGKGGAKR (aa 4-17) were displayed in Additional file 1: Figure S1A and B. Both peptides were detected in multiple PTMs forms, 15 forms for KSAPATGGVKKPHR and 16 for GKGGKGLGKGGAKR, respectively. The retention times and peak areas of these two histone peptides in multiple PTMs forms were extracted under the curve in mass spectra. The histone peptide with multiple PTMs was quantified in relative abundance, the percentage of the area of this histone peptide with particular PTM in summed total area of the histone peptide in all forms [27–29]. Since proteins must be digested into small fragments for mass spec analysis, the long distance multiple PTMs cannot be identified with the approach.

A total of 184 histone peptides including 139 peptides carrying one or more modifications were analyzed (Additional file 2: Table S1). A total of 96 single histone marks were characterized, including 36 marks located in histone H3, 8 in H4, 39 in H2A/B, and 13 in H1 (Fig. 1b and Additional file 2: Table S1). For histone H3 and H4 which are of most interests due to the high occupancy of multiple PTMs on the N-terminal tails, 64 H3 peptides and 23 H4 peptides were quantified, respectively. The relative abundances of histone H3 and H4 peptides in all modified forms were listed in Additional file 2: Table S2. The H3 and H4 peptides generated in this study were found to carry single and co-existing PTMs (Additional file 1: Figure S2A). The abundances of single marks on histone H3 and H4 were calculated by summing the percentages of this modification in all peptide forms and then compared between tumor and normal as listed in Additional file 1: Figure S2B and Additional file 2: Table S3. A heat map representing all the 44 single marks on histone H3 and H4 across all biological replicates under both conditions was illustrated in Fig. 1c. Good reproducibility between biological replicates was obtained and an obvious difference between normal and tumor breast tissues was clearly displayed. The volcano plot made by plotting the fold changes and statistical *p* values further confirmed the significant changes of individual histone single marks in tumor (Fig. 1d). Detailed information for the changes in relative abundances of single marks on H3 and H4 was illustrated in the histogram in Additional file 1: Figure S2B. The abundances of most acetylations appeared to increase in tumor tissues, including H3K4ac, H3K18ac, H4K8ac, H3K27ac, and H4K12ac. Meanwhile, methylations showed diverse regulation. H3K4me3 and H3K36me1/me2 showed a significant increase in tumor, whereas H3K23me1, H3K36me3, and H4K20me3 decreased in tumor.

### The global level of histone modifications verified by western blot

To confirm the above results, we performed western blotting of each identified modification in cancer and normal breast tissues. We did not get good antibodies for some modifications, such as H3K4ac, and then we have to just verify some of the altered single modifications (Fig. 1e). The increase of H4K8ac in tumor was confirmed with two different antibodies, and H4K12ac and H3K18ac also exhibited an obvious increase. H3K23ac increased in tumor tissues, while H3K23me1 decreased. H3K27ac only increased slightly, but we kept observing it in multiple repeats. H3K9ac did not change obviously while H3K9me2 seemed to increase in tumor. H3K36me3 also decreased in tumor. Since the peptides for H3K4 modifications are too short, their mass spec results usually not very reliable. Also, considering the difference between the nature of two techniques, some discrepancies were expected. The trends of H4K8ac, H4K12ac, H3K18ac, H3K23me1, H3K23ac, H3K27ac, and H3K36me3 were similar to the proteomic results, confirming these modifications probably did change during tumorigenesis.

### Epigenomic analysis showed the high correlation between H3K27ac and H4K8ac

To further investigate the dynamic chromatin distribution of the above modifications, we performed ChIP-seq analysis. We did not find ChIP-grade antibodies for H3K18ac, H4K12ac, H3K23me1, and H3K23ac, so we only studied the genome localization of H3K27ac, H4K8ac, H3K4me3, and H3K27me3. Here, we used H3K4me3 and H3K27me3 as controls for active and negative gene transcription. The tumors from three different mice were analyzed. For the group of normal tissues, since the tissue amount from one mouse were not enough, we collected and mixed the tissues from three mice for one analysis, and totally three biological replicates were studied.

**Fig. 2:**
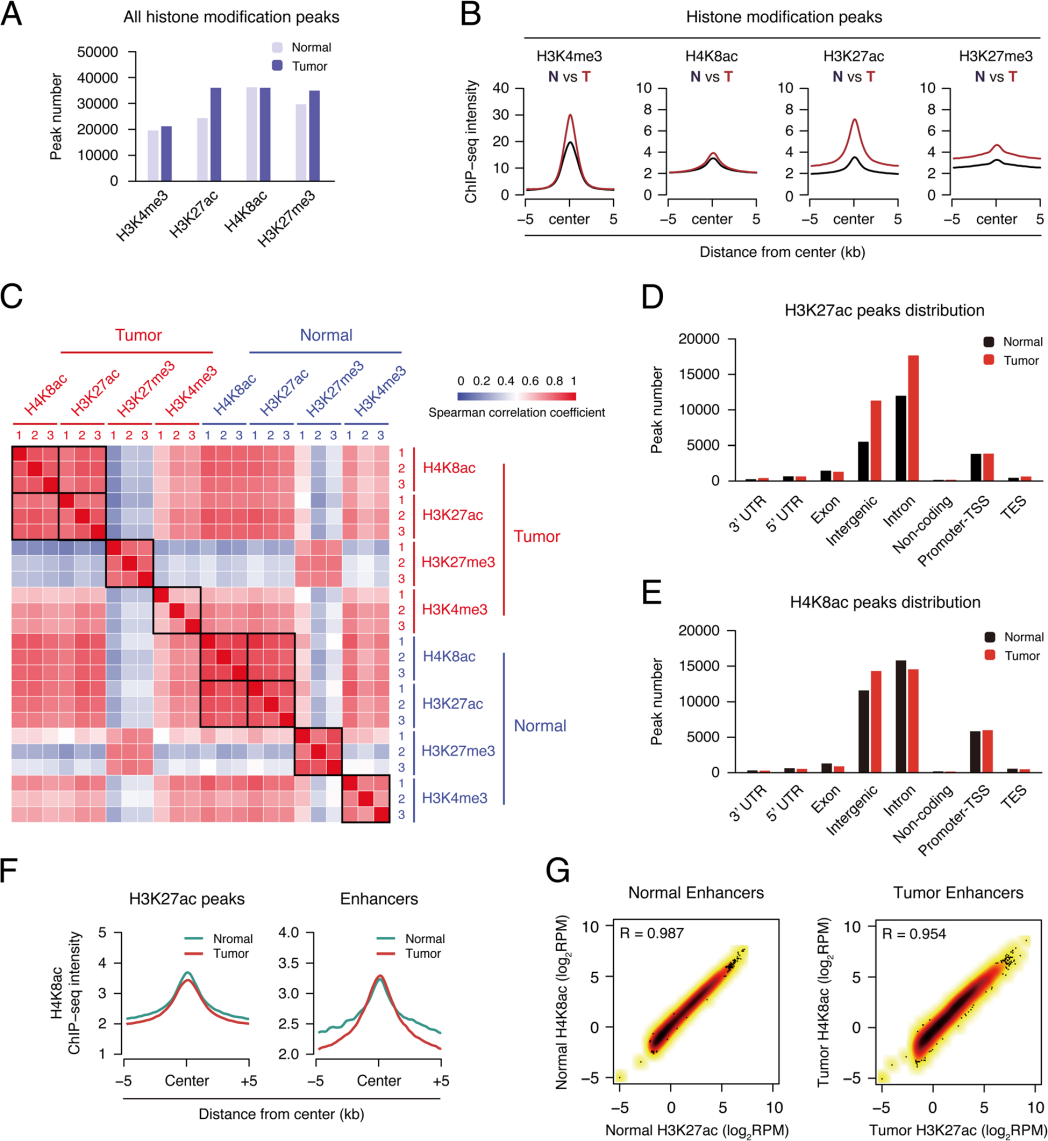
H4K8ac has a high correlation with H3K27ac in the position of the enhancer. **a** Bar plot showing the number of significant peaks from four histone modifications (H3K4me3, H3K27ac, H4K8ac, and H3K27me3) in both normal tissue and tumor. **b** Metagene plot representation of the mean ChIP-seq signal for four indicated histone marks across the individual peaks of each mark. Metagene analysis is centered on the middle of peaks and 10 kb around the center of peaks are displayed (5 kb upstream and 5 kb downstream). Each ChIP-seq data of histone marks were merged by their three individual replicates. **c** Heat map representation of correlation based on four histone modifications (H3K4me3, H3K27ac, H4K8ac, and H3K27me3) occupancy at mouse genome wide. Sample correlations were calculated by Spearman correlation coefficient. **d**, **e** Distribution of histone mark H4K8ac and H3K27ac in different genome elements according to the location of their peaks. Promoter-TSS, “− 1kb to + 100bp” of the transcription start sites. TES, “− 100bp to + 1Kb” of transcription termination sites. **f** Metagene plot representation of the mean H4K8ac signal across H3K27ac peaks and enhancer in both normal tissue and tumor. **g** Scatter plots showing the correlation of H3K27ac and H4K8ac levels in both normal tissue and tumor enhancer. Correlation was calculated by the Pearson correlation coefficient

Our analysis indicated the total peak number of H3K27ac increased in tumor tissues, but that of the other three did not change obviously (Fig. 2a). The signal intensity of all four modifications all increased in tumor, though H4K8ac only increased a small amount (Fig. 2b). Their trends seemed not fully matching the proteomic results and we guessed it was probably due to the use of different normalization standards, input DNA for ChIP-Seq, and total histones for proteomics. Nevertheless, both H3K27ac and H4K8ac increased in tumor tissues, which is similar to the proteomic results.

We then studied the association of chromatin distribution among four modifications in the normal and tumor groups. H3K27me3 was very different from others as expected, while H3K4me3 is quite similar to histone acetylation, since they are all marks for active transcription (Fig. 2c). Interestingly, H3K27ac and H4K8ac showed very high correlation, suggesting their sites were highly overlapped (Fig. 2c). The chromatin distributions of H3K27ac and H4K8ac peaks were similar, both enriched in intergenic regions and introns, and their peak numbers both increased in the intergenic regions in tumor tissues (Fig. 2d, e), suggesting a potential relationship between the change of H3K27ac and H4K8ac in the intergenic regions.

### H3K27ac and H4K8ac are both enriched on distal enhancers

We then analyzed the H4K8ac signal on H3K27ac regions and found that H4K8ac peaked at the center of the global H3K27ac regions, as well as the distal enhancers (Fig. 2f). The further study indicated that H3K27ac and H4K8ac were highly correlated at the genome-wide scale in both normal and tumor tissues (Additional file 1: Figure S3A and B). When looking at the enhancer regions, the correlation of the two modifications was even higher (Fig. 2g). To further support our finding, we downloaded the ChIP-seq data in IMR-90 cells from ENCODE (https://www.encodeproject.org/, or GEO Acc. GSE16256), and the analysis also indicated that H3K27ac and H4K8ac were correlated genome-widely and higher on enhancers (Additional file 1: Figure S3C). All these indicated that H4K8ac is highly correlated with H3K27ac, especially on enhancers, which suggests H4K8ac can perhaps be used as another marker for enhancers.

### Hyper-activation of super-enhancers in the breast cancer tissues

**Fig. 3:**
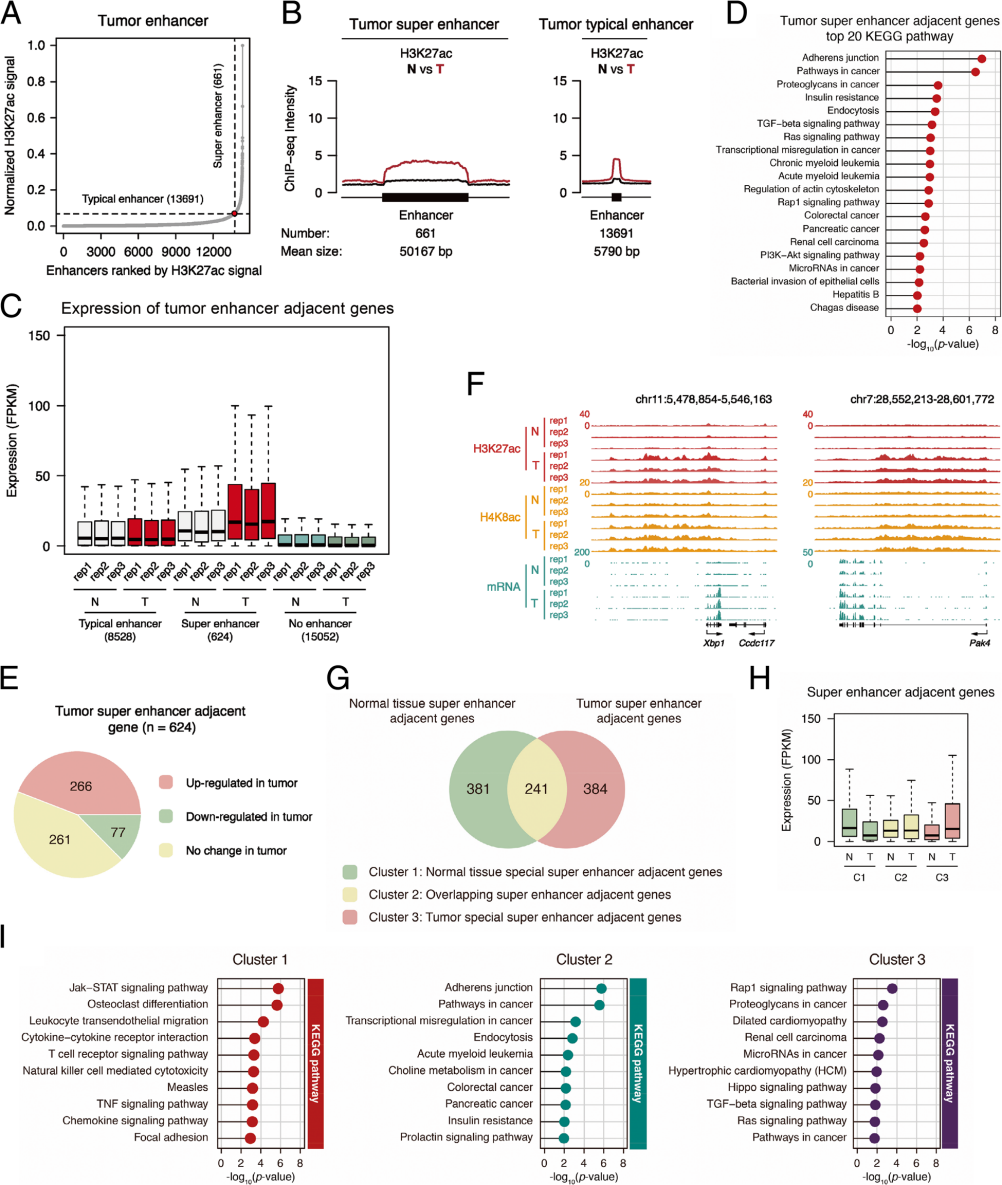
Super-enhancer in tumor involved in breast cancer tumorigenesis. **a** Distribution of H3K27ac ChIP-seq signal (normalize by ratio to highest H3K27ac reads number of enhancer) across the 14,352 tumor enhancers. **b** Metagene analysis of mean H3K27ac ChIP-seq density across 661 super-enhancers and 13,691 typical enhancers. Metagene plot is centered on the enhancer region. The number and mean size of super and typical enhancers are shown. **c** Box plot of average expression of genes (FPKM) adjacent to tumor typical enhancers and super-enhancers, and genes without enhancer, in normal and tumor tissues. **d** Matchstick plot showing the top 20 KEGG pathway associated with tumor super-enhancer-adjacent genes. **e** Pie plot showing the gene number of the three tumor super-enhancers adjacent gene clusters, which are increased in tumor, decreased in tumor, and no change in tumor compared to normal tissue. **f** UCSC browser view showing the ChIP-seq density of H3K27ac, H4K8ac, and RNA-seq signal in both normal tissue and tumor located in Xbp1 and Pak4 gene locus. **g** Venn diagram showing the gene number of the three different clusters indicated in the bottom. **h** Box plot of expression (FPKM) from super-enhancer adjacent genes of the three clusters illustrated in Fig. 3g. **i** Matchstick plots showing the top KEGG pathway terms of the three gene clusters identified in Fig. 3g

The above data indicated that H3K27ac and H4K8ac are higher in tumor, which suggests the enhancer activity in tumor was potentially higher. The early study found that super-enhancers in cancer cells are enriched at oncogenes and others important in tumor pathogenesis. [23]. So, we analyzed the potential roles of super-enhancers in our model. We first identified the super and typical enhancers in the tumor and normal tissues according to the previous description [22] (Fig. 3a and Additional file 1: Figure S4A). On the super and typical enhancers identified in tumor, H3K27ac level was much higher in tumor than that in normal tissues (Fig. 3b). We hypothesized that the adjacent genes represent their target genes. The expression of genes adjacent to tumor super-enhancers increased more than those adjacent to typical enhancers and others (Fig. 3c), and the genes adjacent to tumor super-enhancers were enriched in cancer-related pathways (Fig. 3d). Totally, 624 genes were identified adjacent to tumor super-enhancers, among which 266 genes expressed higher in tumor tissues (Fig. 3e). Two representative genes, Xbp1 and Pak4, were shown in Fig. 3f. As a comparison, the genes adjacent to super-enhancers of normal tissues did not express more in tumor (Additional file 1: Figure S4B). The adjacent genes of tumor super-enhancers, compared with those unique in normal tissues, were more related with cancer (Fig. 3g–i). These data are consistent with the previous report that the activation of super-enhancers represents tumor characteristics [23]. Our analysis confirmed the previous hypothesis about the role of super-enhancers in cancer and identified a group of super-enhancers important for cancer growth and development in the current model (Additional file 2: Table S4).

### The elevation of H3K27ac and H4K8ac in the patient breast cancer tissues

**Fig. 4:**
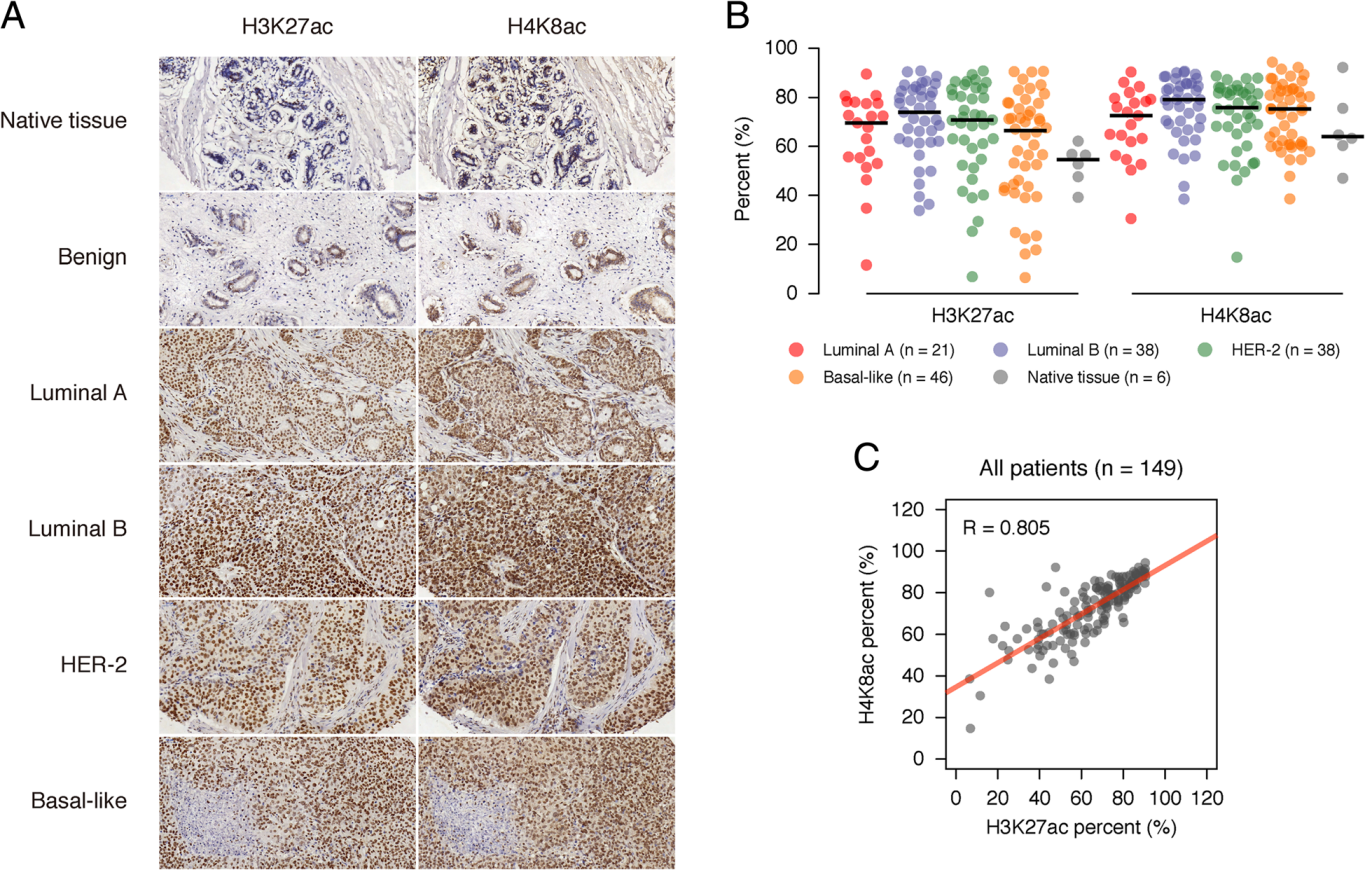
H3K27ac and H4K8ac were highly enriched in clinical breast cancer patients. **a** IHC examples representation of H3K27ac and H4K8ac enrichment levels in native tissue, benign as well as four breast cancer sub-types from clinical patients. **b** Dot plot showing the H3K27ac and H4K8ac enrichment levels in four breast cancer sub-types and native tissue according to clinical patient IHC arrays. The black line indicates the median value of a group. **c** Scatter plot illustration of H3K27ac and H4K8ac enrichment levels according to clinical patient IHC arrays. The matching line was labeled by the red solid line. Samples correlation were calculated by the Pearson correlation coefficient

Then, we investigated whether our finding about H3K27ac and H4K8ac is true in human patient breast tumor tissues. We purchased two sets of commercialized slides, which were accompanied with tumor molecular information. IHC staining was performed with H3K27ac and H4K8ac antibodies, respectively. Consistent with our previous data, the two modifications were low in the native and benign tissues and increased obviously in all types of breast cancer tissues (Fig. 4a). We scanned the slides with Leica Aperio VERSA 8 digital pathology scanner. The results indicated that both H3K27ac and H4K8ac are higher in cancer than normal tissues, highest in luminal B type (Fig. 4b). The two marks also showed very high correlation in the patient tissues (Fig. 4c), supporting our previous results.

### Identification of H3K4me3 super-enhancers in breast cancer tissues

**Fig. 5:**
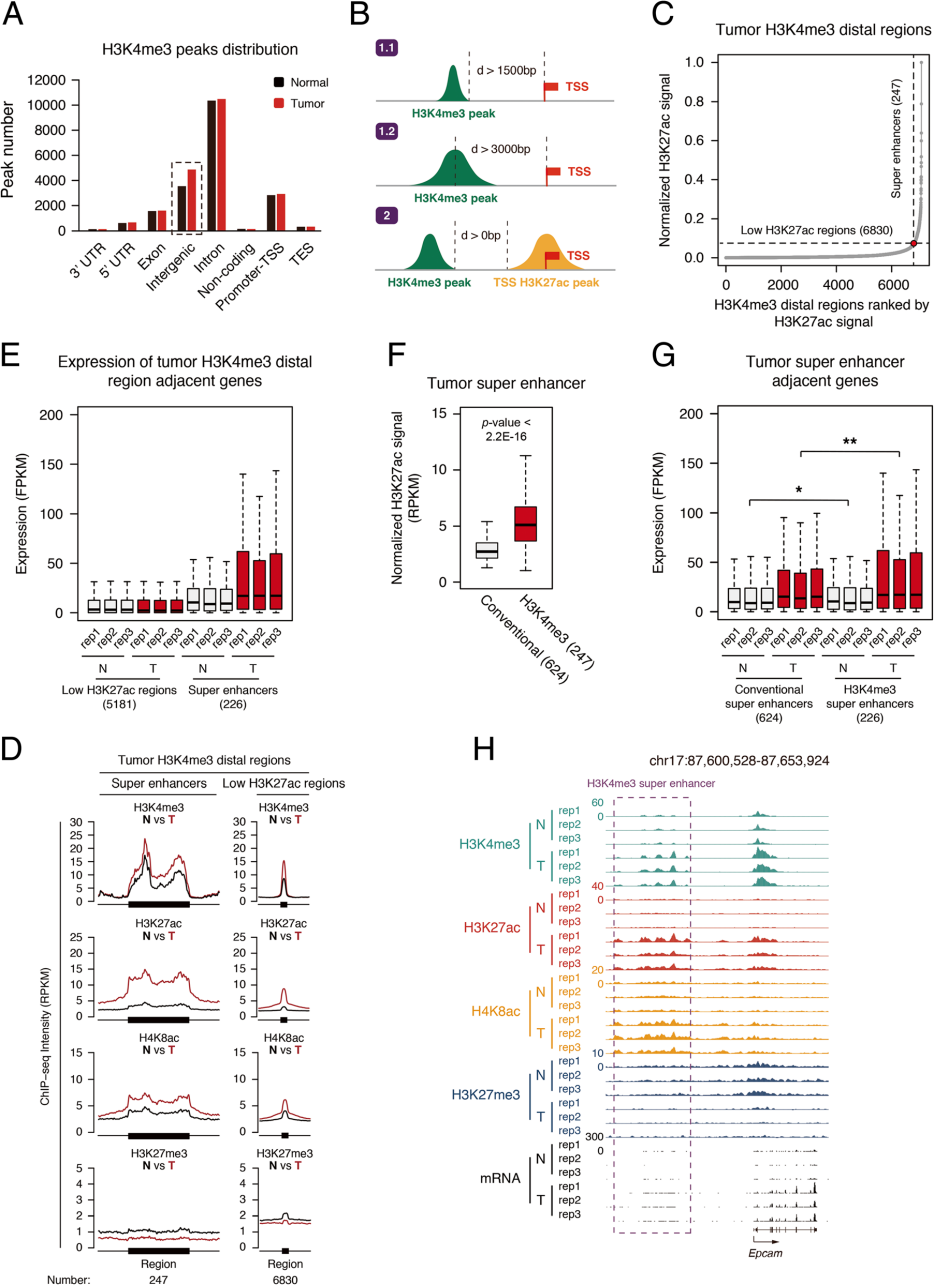
Identification of H3K4me3-enriched super-enhancers. **a** Distribution of histone mark H3K4me3 in different genome element according to the location of their significant peaks. Promoter-TSS, “− 1kb to + 100bp” of the transcription start sites. TES, “− 100bp to + 1Kb” of transcription termination sites. **b** Sketch of two methods identifying the H3K4me3 peaks away from the TSS region. **c** Distribution of H3K27ac ChIP-seq signal (normalize by ratio to highest H3K27ac reads number of enhancer) across the 4048 tumor H3K4me3-enriched enhancers. **d** Metagene analysis of mean histone marks H3K4me3, H3K27ac, H4K8ac, and H3K27me3 ChIP-seq density across tumor H3K4me3-enriched super and typical enhancers. Metagene plot is centered on the enhancer region. The number and mean size of super and typical enhancers are shown. **e** Box plot of expression (FPKM) from tumor H3K4me3-enriched typical and super-enhancer adjacent genes in both normal tissue and tumor. **f** Box plot of expression (FPKM) from traditional and H3K4me3-enriched super-enhancer adjacent genes in tumor. *p < 0.05, **p < 0.01. **g** UCSC browser view showing the ChIP-seq density of H3K4me3, H3K27ac, H4K8ac, H3K27me3, and RNA-seq signal in both normal tissue and tumor located in Epcam gene locus

From the analyzed data, we noticed that the peak number of H3K4me3 in intergenic regions increased in tumor tissues, but not any other regions (Fig. 5a). Recent studies reported the intergenic H3K4me3 also marks enhancers [30, 31]. We speculated that the increased H3K4me3 peaks may represent a subclass of enhancers in tumor tissues and analyzed the H3K4me3-marked enhancers in our model. We defined the H3K4me3 peaks whose covered regions were more than 1 .5 kb away or whose centers were more than 3 kb away from TSS as distal H3K4me3 peaks (Fig. 5b). We identified all the distal H3K4me3 peaks and merged the peaks within 12.5 kb together, and then we ranked H3K27ac of the regions and got a plot similar to what we did for conventional super-enhancers. A tangent line with slop 1 was drawn and those higher than the intersection point were defined as H3K4me3 super-enhancers. Totally, 293 H3K4me3 super-enhancers in tumor tissues were identified (Fig. 5c and Additional file 2: Table S5). Using the same strategy, we identified 225 H3K4me3 super-enhancers in normal breast tissues (Additional file 1: Figure S5A).

### The H3K4me3 super-enhancers were associated with higher transcription activity

Then, we took the groups of tumor H3K4me3 enhancers and analyzed their modifications and related gene transcription. The H3K4me3 super-enhancers showed higher H3K4me3 level in tumor than that in normal tissues, and so did H3K27ac and H4K8ac; while H3K27me3 behaved oppositely (Fig. 5d). Their adjacent genes also expressed higher in tumor than normal tissues (Fig. 5e). It is not surprising since H3K4me3 super-enhancers are identified similar to conventional super-enhancers. Interestingly, the H3K27ac signal in the H3K4me3 regions of H3k4me3 super-enhancers is much higher than the average level of conventional super-enhancers (Fig. 5f). The adjacent gene expression of tumor H3K4me3 super-enhancers was higher than tumor conventional super-enhancers (Fig. 5g). These suggest H3K4me3 super-enhancers are more active than conventional super-enhancers. EPCAM is a carcinoma-associated antigen which often highly expresses in tumor cells. We used it as a sample to show its higher expression and increased H3K4me3, H3K27ac, and H3K8ac on its enhancer (Fig. 5h). We noticed that the RNA signal at its enhancer region also increased in tumor, which may represent the upregulation of its eRNA (Fig. 5h). As a comparison, on the H3K4me3 enhancers of normal tissues, H3K4me3 did not increase significantly in tumor and has less elevation of H3K27ac (Additional file 1: Figure S5B). The related gene expression did not increase in tumor either (Additional file 1: Figure S5C). Taken together, these suggest that the increased H3K4me3 super-enhancers in tumor cells probably causes elevated transcriptional activity. Moreover, H3K4me3-marked enhancers are not limited to cancer tissues, and our analysis of online data shows that intergenic H3K4me3 peaks at the center of enhancers in IMR-90 cell lines (Additional file 1: Figure S5D).

### H3K4me3 super-enhancers were associated with cancer-related genes

**Fig. 6:**
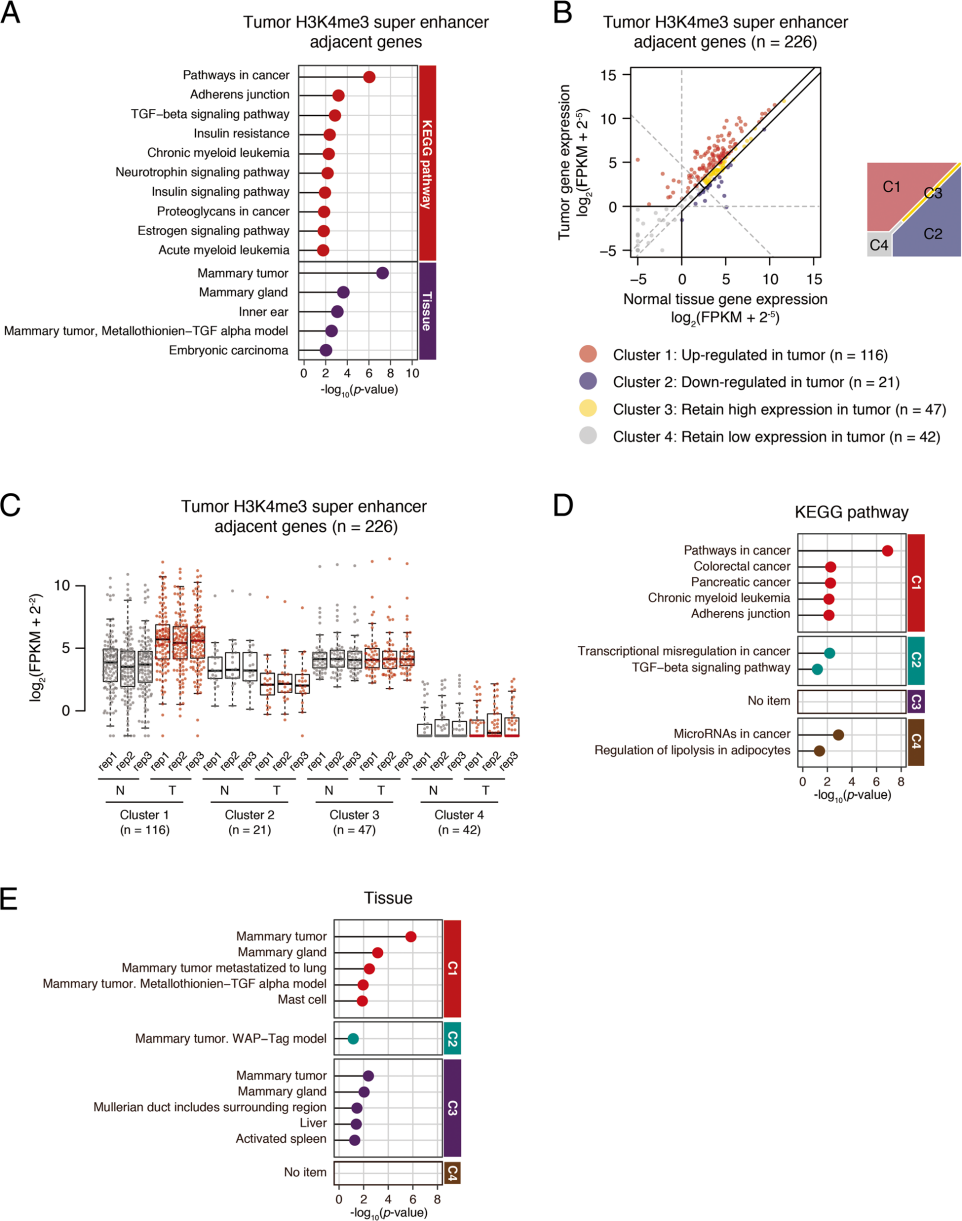
Genes associated with H3K4me3-enriched super-enhancer were highly related to breast cancer tumorigenesis. **a** Matchstick plot showing the top 10 KEGG pathway and top 5 specific expressed tissue associated with tumor H3K4me3-enriched super-enhancer-adjacent genes. **b** Scatter plot comparing tumor H3K4me3-enriched super-enhancer-adjacent genes expression (log_2_(FPKM + 2^−5^)) between normal tissue and tumor. All 275 genes were divided into four clusters. **c** Box plot of expression (log_2_(FPKM + 2^−2^)) from four gene clusters identified in 5C in both normal tissue and tumor three replicates. **d**, **e** Matchstick plots showing the top KEGG pathway (**e**) and specifically expressed tissue (**f**) terms of four gene clusters identified in 5C

After we knew that tumor H3K4me3 super-enhancers are associated with increased transcription activity, we then analyzed whether their functions are associated with cancer progression. The KEGG pathway analysis showed that the adjacent genes to tumor H3K4me3 super-enhancers are not only enriched in cancer pathways, but also in some breast mammary pathways (Fig. 6a). If we divided these genes into four groups according to their expression change (Fig. 6b, c), the genes with upregulated expression in tumor (cluster 1) were associated with cancer pathways, especially breast cancer. Other genes were mostly associated with metabolic pathways and others (Fig. 6d, e). We chose some genes in cluster 1 and confirmed their expression and chromatin state with experiments (Additional file 1: Figure S6). These inform us that it is useful to combine the enhancer analysis with gene expression profiling to identify key genes in tumorigenesis and metastasis.

### Inhibition of p300 repressed tumor growth in mice model

**Fig. 7:**
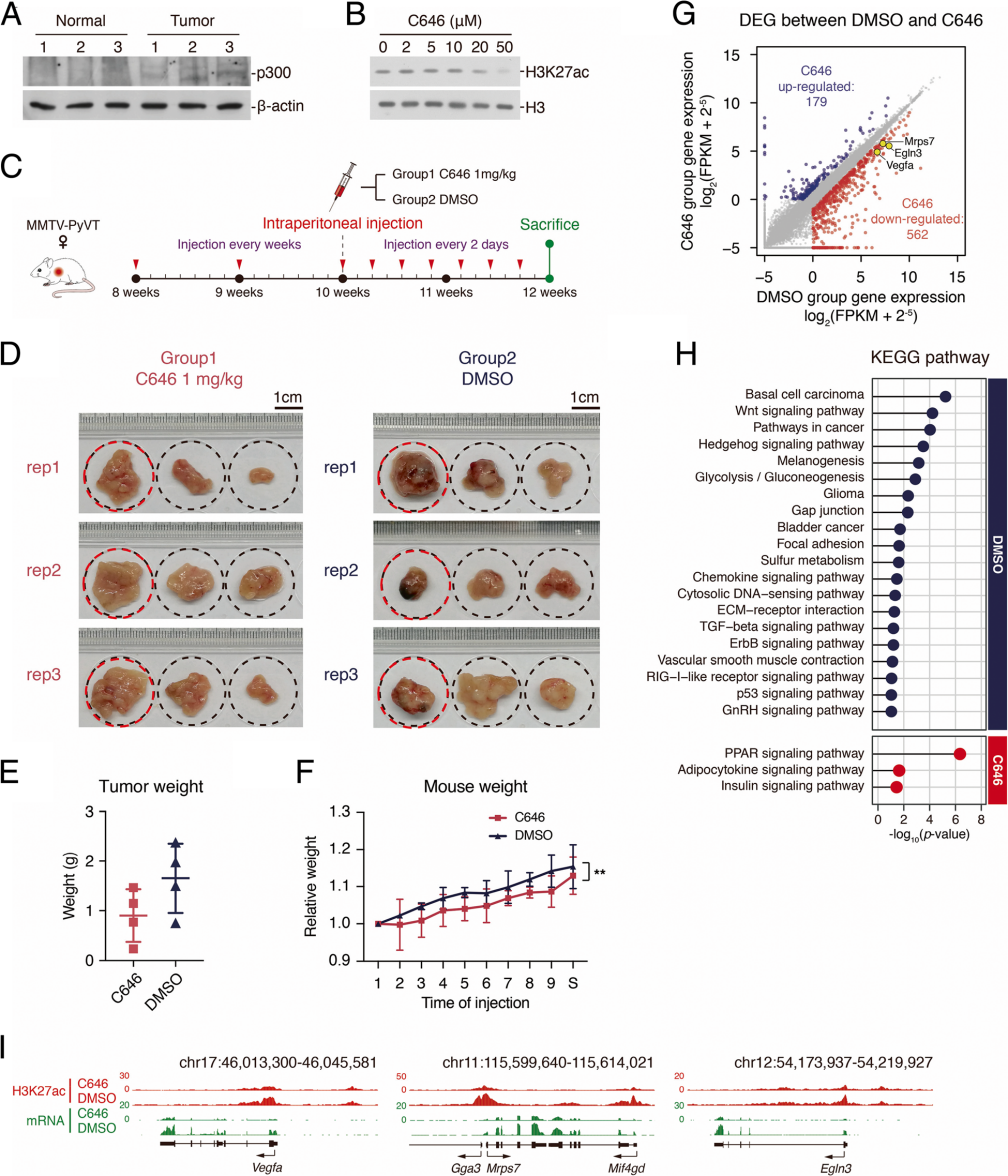
p300 inhibitor C646 could suppress breast cancer development in MMTV-PyVT mice. **a** Western blot analysis with p300. Whole tissue lysates were prepared from normal tissue or tumor three replicates. **b** Western blot analysis with H3K27ac after adding p300 inhibitor C646 in different concentration (0, 2, 5, 10, 20, 50 μM) to HEK293 cell line. Cell was treated with C646 for 12 h. **c** Sketch showing the schedule of C646 injection. **d** Photos of MMTV-PyVT mouse mammary tumors grown 12 weeks. The successive three tumors in a rectangle photo belong to one female MMTV-PyVT mouse, and they were the biggest three tumors in the mouse. All dashed circles represent the same size and the scales locate in the top right corner of each group. The tissues with red circles were subjected for RNA-seq. **e** Scatter plot showing the tumor weight of the two groups. Summation weight of the biggest three tumors was considered as the tumor weight of each mouse. **f** Line plot showing the body weight development of mouse in the two groups. **p < 0.001; *t* test; *n* = 4. **g** Scatter plot comparing upregulated genes expression (log_2_(FPKM + 2^−5^)) between DMSO group and C646 group. **h** Matchstick plots showing the top 20 (top) and all three (bottom) KEGG pathway terms of DMSO group and C646 group upregulated genes. **i** UCSC browser view showing the ChIP-seq density of H3K27ac and RNA-seq signal in both DMSO group and C646 group located in *Vegfa*, *Mrps7*, and *Egln3* gene locus

Next, we started to think whether we can repress tumorigenesis through controlling enhancer activity. Unfortunately, we could not find a chemical inhibitor for H3K4me3 on enhancer. Then, we tried to manipulate the level of H3K27ac in the cell. When investigating the molecular mechanisms for enhancer regulation in the tumor, we found the protein level of p300 increased in tumors (Fig. 7a). Since p300 is the known enzyme for H3K27ac on enhancers, and it also catalyzes acetylation on H4K8, we thought the increased p300 may be responsible for the elevation of H3K27ac and H4K8ac in tumor. We then utilized one reported inhibitor for p300, C646 [32], and found that the treatment of C646 to HEK293 inhibited H3K27ac, indicating the drug is functional (Fig. 7b). Then, we applied it to the MMTV-PyVT mice model (Fig. 7c). After C646 treatment, some of the tumor-like tissues were still formed. Morphologically, the tissues were flatter and had no dark blood vessels compared with the DMSO control group (Fig. 7d). The average tumor weight was less after C646 treatment (Fig. 7e), but the HE staining showed no difference of cell morphology between two groups (data not shown). The average mice weight also decreased a little bit, maybe because of the drug toxicity (Fig. 7f). These data suggest that C646 treatment inhibited some cancer features in the current condition.

### P300 inhibition selectively inhibits the expression of oncogenes in breast tumors

We then performed RNA-seq and H3K27ac ChIP-seq analysis with the collected tumor tissues. The biggest tumor tissues of the three mice for each group were analyzed. The analysis of global gene expression showed that the different expressed genes (DEGs) highly expressed in DMSO group were largely enriched in cancer-related pathways, while those highly expressed in C646 group were less related (Fig. 7g, h). From the DEGs, we found that *Vegfa* was downregulated and its H3K27ac decreased after C646 treatment (Fig. 7i). Since *Vegfa* is a well-known gene to regulate angiogenesis, C646 probably inhibits tumor growth through *Vegfa* and other cancer-related genes.

## Discussion

In the current study, our proteomics and western blotting results revealed that histone H3K27ac and H4K8ac are upregulated in the tumors of MMTV-PyVT mice, which was confirmed by IHC staining of human breast cancer tissues. Our ChIP-seq analysis further discovered H4K8ac is largely co-localized with H3K27ac on chromatin, especially on distal enhancers; thus, H4K8ac may serve as another novel mark for enhancer. The enhancer profiling in the mouse tumor further found that H3K4me3 marks a group of distal enhancers and the H3K4me3 super-enhancers may become one of the important features for cancer which is coupled with higher expression of cancer-related genes. These analyses provide important proteomic and epigenomic resource for breast cancer studies. Though the intergenic regions of mouse are quite different from human, the enhancer profiling will still be useful to the future mechanistic studies in mice.

Increased activity of super-enhancers has been associated with tumorigenesis, and the super-enhancers and their target genes are considered as potential markers for tumor cell identity [5, 23]. Here, we not only identified the super-enhancers specific in the tumors of mice model, but we also identified a novel subclass of super-enhancers which are marked by H3K4me3. H3K4me3 super-enhancers are associated with higher transcription level of their adjacent genes than the conventional super-enhancers, and their H3K4me3-enriched regions are associated with higher H3K27ac and H4K8ac signals. Combined analysis of H3K4me3 super-enhancer with global gene expression identified a group of genes enriched in cancer-related pathways. The approach should be helpful to pinpoint the critical enhancers and target genes and may be useful for the future epigenome-wide association study (EWAS) in patient tumors.

A recent publication has raised the concern about the quality of H3K4me3 ChIP-seq [33]. To ensure the quality of our data, we used three H3K4me3 antibodies from a different company and verify some enhancers identified in our study (Additional file 1: Figure S7). The results showed a very similar pattern for all three antibodies. Moreover, we downloaded the analyzed data performed with ICeChIP and good antibodies and compared the peak number, ratios, and shapes among these datasets. The results showed that our data with EMD 04-745 have a similar pattern as theirs (Additional file 1: Figure S8).

Our study reveals that the genome-wide distributions of H4K8ac and H3K27ac are largely overlapped, especially on enhancers. The histone acetylation transferases (HAT) often contain enzymatic activity to multiple histone sites; however, after analyzing the ChIP-seq data in ENCODE database, we found that the chromatin distributions of the acetylation on different sites seem to be somehow overlapped but not identical (data not shown). These raise the question whether the combination of different histone acetylation has certain biological functions? For example, selectively regulating gene transcription or contributing to genome structure? The comprehensive study of multiple histone acetylations across the genome will be required to answer it.

Besides H4K8ac and H3K27ac, we have also identified other modifications through both mass spec and western blotting, such as H3K18ac, H3K23ac, H4K12ac, H3K23me1, and H3K36me3. Unfortunately, due to lacking high-quality ChIP-grade antibodies or not enough resource, we did not pursue for their functions in tumorigenesis and development. It will be interesting to further investigate their functions and verify them in patient tissues.

We found that H4K8ac and H3K27ac are upregulated in breast cancer tissues. We also applied one inhibitor of p300, C646, to modulate the activity of enhancers in the tumor model. Though C646 did not completely inhibit tumor formation, it did significantly repress tumor formation through altering the gene expression program, especially the angiogenesis process through inhibiting *Vegfa* expression. Further effort to find better p300 inhibitors or the combinational use with other drugs may be promising as novel strategies for drug development.

## Conclusions

The different histone modifications between breast cancer and normal tissues were studied. We found that H3K27ac and H4K8ac significantly in cancer and confirmed it with patient tissue chips. H4K8ac is co-localized with H3K27ac on distal enhancers. H3K4me3 marks a subgroup of super-enhancers in the intergenic regions, which are associated with higher level of H3K27ac and H4K8ac, as well as higher transcription level of their adjacent genes. One hundred sixteen H3K4me3-enriched super-enhancers with higher gene expression in tumor were identified. C646, an inhibitor of H3K27ac, repressed tumor formation through inhibiting the expression of *Vegfa* and other genes. Taken together, we have identified novel regulators, provide important resource to the genome-wide enhancer studies of breast cancer, and raise the possibility of cancer treatment through modulating enhancer activity.

## Methods

### Aim and design of the study

The aim was to study the difference of histone modifications in breast cancer tissues compared with normal tissues in the global scale. Cancer tissues and corresponding normal tissues were collected. Mass spec of histone modifications was performed and confirmed with western and patient tissue chips. Then, ChIP-seq study was performed to identify the different enhancer sites. Finally, an inhibitor of H3K27ac, which represent enhancer activity, was applied to test its effect on cancer.

### Materials and reagents

Antibodies, H3K4me3 (clone MC315, Millipore 04-745), H3K27ac (Abcam Ab4729), H3K27me3 (clone C36B11, CST 9733), H4K8ac (Abcam Ab45166 for ChIP-Seq, IHC and western, Abclonal A7258 for western), H4K12ac (CST 13944), H3K27me1 (clone D3R8N, CST 84932), H3K23ac (CST 14932), H3K23me1 (Active motif, 39388), H3K9ac (clone C5B11, CST 9649), H3K9me2 (Abclonal A2359), H3K9me3 (Abcam ab176916), H3K4me1 (CST 5326), H4K20me1 (Abcam ab9051), H3K36me3 (Abcam ab9050), H4K20me3 (Abclonal A2372), H3 (Abcam Ab1791), p300 (Abcam ab14984), and β-actin (Abclonal AC026), were purchased from the indicated merchants.

### Mice feeding and tissue collection

FVB/N-Tg(MMTV-PyVT)634Mul/J transgenic mice [34] and wild-type FVB mice were purchased from Nanjing Biomedical Research Institute of Nanjing University (http://www.nbri-nju.com/). All mice were housed in the SPF grade room of Animal Center of College of Life Sciences, Wuhan University. All the animal operations were following the laboratory animal guidelines of Wuhan University and were approved by the Animal Experimentations Ethics Committee of Wuhan University (Protocol NO. 14110B).

Heterozygous female MMTV-PyVT (Mtv/-) mouse was backcrossed onto wild-type male FVB mouse and we selected male MMTV-PyVT (Mtv/-) mice for mating, female MMTV-PyVT (Mtv/-) and female wild-type FVB mice for subsequent experiments. Mammary tumors were gotten from female MMTV-PyVT in 12 weeks old; meanwhile, normal mammary glands from female wild-type FVB of 12 weeks old were taken out. Tumors grown in thoracic mammary gland were selected as the tumor samples, 1 mouse for 1 biological replicate. Abdominal and inguinal mammary glands were selected as the normal tissue samples, 3 mice for 1 biological replicate because of the low amount of tissue in normal mammary gland.

### Tissue microarray and IHC

The tissue microarray slide was purchased from Xi’an Alenabio Co., Ltd. (Cat. NO. BR1503e and BR1505b). The BR1505b slide contained 75 cases (150 cores) of breast invasive ductal carcinoma samples. The BR1503e slide contained 3 cases (6 cores) of adjacent normal breast tissue, 3 cases (6 cores) of breast fibroadenoma samples, 2 cases (4 cores) of breast cystosarcoma phyllodes samples, 7 cases (14 cores) of breast intraductal carcinoma samples, and 60 cases (120 cores) of breast invasive ductal carcinoma samples.

The slides were deparaffinized, rehydrated, and subjected to heat-mediated antigen retrieval. For immunohistochemistry (IHC) analysis, the sections were incubated with 3% H2O2 for 15 min at room temperature to quench endogenous peroxidase activity. After incubating in normal goat serum for 1 h, sections were treated with primary antibody at 4 °C overnight. IHC analysis of tumor samples was performed using primary antibodies against H4K8ac (dilution 1:200; Abcam ab45166) and H3K27ac (dilution 1:200; Abcam ab4729). The sections were then washed three times in PBS and treated for 30 min with biotinylated goat-anti-rabbit IgG secondary antibodies. After washing three times in PBS, sections were incubated with streptavidin-conjugated HRP. After washing three times in PBS for 5 min each, specific detection was developed with 303-diaminobenzidine (DAB-2031). Images were taken by Leica Aperio VERSA 8 digital pathology scanner.

For information about other experimental methods, please refer to the supplemental materials.

### Molecular subtypes of breast cancer

Molecular subtypes of breast cancer were determined as follows: Luminal A—breast cancer is hormone-receptor positive (estrogen-receptor and/or progesterone-receptor positive), HER-2 negative, and has low levels of the protein Ki-67. Luminal B—breast cancer is hormone-receptor positive (estrogen-receptor and/or progesterone-receptor positive) and either HER-2 positive or HER-2 negative with high levels of Ki-67. HER-2-type breast cancer is hormone-receptor negative (estrogen-receptor and progesterone-receptor negative) and HER-2 positive. Basal-like breast cancer is hormone-receptor negative (estrogen-receptor and progesterone-receptor negative) and HER-2 negative.

### IHC image analysis

The digital slides were taken by Leica Aperio VERSA 8 digital pathology scanner and then analyzed by Aperio colocalization image analysis algorithm. First, we use ImageScope to view the digital slides and select the appropriate area to calibrate the color and threshold of hematoxylin and DAB. Then, we create an algorithm macro and register it on the eSlide Manager. The eSlide Manager provides a convenient tool for batch analysis of slides. We used the selected algorithm macro to analyze the digital slides. The percent of DAB represented the protein concentration.

### Histone extraction for LC-MS/MS analysis

Mouse breast tissues were minced and homogenized on ice in 1:10 (*wt*/*v*) NIB buffer within 10 mM sodium butyrate, 1 × protease inhibitor cocktail, 1 × phosphatase inhibitor cocktail, and 0.2% NP-40. After homogenization for 10 min, the nuclei were pelleted by centrifugation for 5 min at 1000*g*. The nuclei samples were washed gently three times in 1:10 (*v*/*v*) NIB buffer without 0.2% NP-40. Histones were then extracted from the nuclei in 1:5 (*v*/*v*) 0.2 M H2SO4 for 3 h and precipitated by 33% TCA on ice overnight. Histones were washed using ice-cold acetone supplemented with 0.1% HCl and then ice-cold acetone without 0.1% HCl, respectively. Histones were dried in a vacuum centrifuge. The concentration of extracted histone was measured by the bio-rad protein assay. The purity and quality of histones were affirmed by 15% SDS-PAGE analysis and Coomassie staining.

### Proteomics studies of histone modifications

#### Buffer preparation

The buffer was prepared with the following: HPLC-grade acetonitrile (ACN) (Millipore), propionic anhydride (Sigma), sequencing-grade modified trypsin (Promega), HPLC buffer A:0.1% formic acid (Sigma), and HPLC buffer B: 0.1% formic acid in ACN.

#### Chemical derivatization and digestion of histones

Twenty micrograms of histone proteins were redissolved in 30 μl 50 mM NH_4_HCO_3_. The lysine residues and N-terminus of histone were chemically derivatizated twice by propionic anhydride with 1:3 (*v*/*v*) acetonitrile at 37 °C and completely dried. Histone proteins were then re-suspended in 30 μl 50 mM NH_4_HCO_3_ and digested by 1:10 (*wt*/*wt*) trypsin at 37 °C overnight. Subsequently, the histone peptides were derivatizated twice with propionic anhydride to label the peptide N-terminus generated from the trypsin digestion and dried completely via a vacuum centrifuge.

#### Stage tip desalting

Stage tips were prepared in the house using double-layered reversed-phase material C18 in 200 μl of tip. Stage tips were washed using 70% ACN/0.1% TFA and balanced using 0.1% TFA. Histone peptides were redissolved in 0.1% TFA and loaded in stage tips twice. The stage tips within histone peptides were washed twice by 0.1% TFA for removing extra salt. Histone peptides were eluted from stage tips using 50% ACN/0.1% TFA and 70% ACN/0.1% TFA and dried in a vacuum centrifuge.

#### LC-MS/MS of histone peptides

Histone peptides were resuspended with HPLC buffer A. Peptides were loaded on to 100 μm i.d. × 2-cm Reprosil-Pur C18-AQ (5 μm; Thermo Scientific, CA) trap column and separated by 75 μm i.d. × 25-cm Reprosil-Pur C18-AQ (2 μm; Thermo Scientific, CA) analytical column. The HPLC gradient was as follows: from 5 to 35% buffer B in 50 min, from 35 to 95% buffer B for 5 min, and 95% buffer B for 5 min at a flow rate of 250 nL/min. NanoLC was coupled to a Q Exactive HF mass spectrometer (Thermo Scientific). For data-independent acquisition (DIA), two full-scan MS spectra (m/z 300–1100) at a resolution of 60,000 were acquired in the orbitrap within a DIA duty cycle, and 16 MS/MS were performed at a resolution of 60,000 with an isolation window of 50 Da. Normalized collision energy (NCE) was set to 27.

#### Data analysis

The raw DIA MS data were processed using Epiprofile, which allowed quantitative analysis of histone peptides with multiple PTMs. Based on previous knowledge of histone peptide masses and elution profiles, the retention times and peak areas of histone peptides were extracted under the curve in mass spectra. A histone peptide with multiple PTMs was quantified in relative abundance, which was the percentage of the area of this histone peptide with particular PTM in summed total area of the histone peptide in all forms. The relative abundance of a single histone mark was obtained by summing the relative abundances in all the modified peptide forms. All quantitative results were expressed as the mean ± SD (standard deviation). For normal and tumor comparison, data were analyzed by homoscedastic *t* test based on biological replicates. Differences were considered statistically significant when the *p* values were less than 0.05.

### Epigenomic studies of histone modifications of mouse tissues

#### Chromatin immunoprecipitation (ChIP) assay of mouse tissues

Sixty milligrams of each tissue was cut into 1-mm^3^ pieces in PBS with protease inhibitor. Tissue pieces were cross-linked for 10 min at room temperature with 1% formaldehyde and then quenched with 0.125 M of glycine for 5 min. Cross-linked tissues were triturated by trituration equipment for 30 s and then centrifuged at 12000 rpm, 4 °C for 5 min. Supernatant with massive oil was discarded and the precipitates were lysed with 1 mL lysis buffer (50 mM Tris-HCl pH 8.0, 0.1% SDS, 5 mM EDTA) and incubated for 5 min with gentle rotation. After centrifugation at 12000 rpm, 4 °C for 2 min, lysates were washed once by digestion buffer (50 mM Tris-HCl pH 8.0, 1 mM CaCl_2_, 0.2% Triton X-100). Washed lysates were incubated in 630-μL digestion buffer with 1-μL MNase (NEB, M0247S) at 37 °C for 20 min and then quenched with 8 μL 0.5 M EDTA. Whole lysates were sonicated and the supernatants were taken out after centrifugation. Thirty microliters of supernatants were taken for checking the efficiency of MNase digestion. Immunoprecipitation was further performed with 150-μL sheared chromatin, 2-μg antibody, 50-μL Protein G sepharose beads and 800-μL dilution buffer (20 mM Tris-HCl pH 8.0, 150 mM NaCl, 2 mM EDTA, 1% Triton X-100, 0.1% SDS) overnight at 4 °C. Next day, immuno-complexes were washed once with Wash buffer I (20 mM Tris-HCl pH 8.0, 150 mM NaCl, 2 mM EDTA, 1% Triton X-100, 0.1% SDS), once with Wash buffer II (20 mM Tris-HCl pH 8.0, 500 mM NaCl, 2 mM EDTA, 1% Triton X-100, 0.1% SDS), once with Wash buffer III (10 mM Tris-HCl pH 8.0, 250 mM LiCl, 1 mM EDTA, 1% Na-deoxycholate, 1% NP-40), and twice with TE (10 mM Tris-HCl pH 8.0, 1 mM EDTA). The immune complexes were eluted twice with 100-μL elution buffer (1% SDS, 0 .1M NaHCO_3_, 20 mg/mL Proteinase K) at room temperature. The elution was incubated at 65 °C for 6 h and then purified with DNA purification kit (TIANGEN DP214-03).

#### ChIP-seq library construction

ChIP-seq libraries were constructed by VATHS Universal DNA Library Prep Kit for Illumina (Vazyme ND604). Briefly, 50 μL of purified ChIP DNA (8–10 ng) was end-repaired for dA tailing, followed by adaptor ligation. Each adaptor was marked with a barcode of 6 bp which can be recognized after mixing different samples together. Adaptor-ligated ChIP DNA was purified by AMPure XP beads (1:1) and then amplified by PCR of 11–13 cycles with the primer matching with adaptor universal part. Amplified ChIP DNA was purified again using AMPure XP beads (1:1) in 35-μL EB elution buffer. For multiplexing, libraries with different barcode were mixed together with equal molar quantities by considering appropriate sequencing depth (30–40 million reads per library). Libraries were sequenced by Illumina Hi-seq X Ten platform with pair-end reads of 150 bp.

#### RNA-seq library construction

For both normal mammary gland and tumor samples, a 20-mg tissue was used for RNA extraction. Total RNA was extracted by using EASYspin RNA Mini Kit (Aidlab RN07). Briefly, tissue was triturated by trituration equipment for 20 s in lysis buffer provided by kit and then centrifuged at 12000 rpm, 4 °C for 5 min. Liquid between precipitates on the bottom and oil on the top was taken out and pipetted 10 times using 1 mL syringe. The entire volume of liquid was added into adsorption column provided by kit and RNA would retain in column while other components include DNA and protein would be washed out by several buffers. Total RNA eluted in 50-μL RNase-free water. RNA-seq libraries were constructed by NEBNext Poly(A) mRNA Magnetic Isolation Module (NEB E7490) and NEBNext Ultra II Non-Directional RNA Second Strand Synthesis Module (NEB E6111). Briefly, mRNA was extracted by poly-A selected with magnetic beads with poly-T and transformed into cDNA by first and second strand synthesis. Newly synthesized cDNA was purified by AMPure XP beads (1:1) and eluted in 50-μL nucleotide-free water. Subsequent procedures were the same as ChIP-seq library construction described previously except the sequencing depth of 20 million reads per library. RNA-seq libraries were sequenced by Illumina Hi-seq X Ten platform with pair-end reads of 150 bp.

#### ChIP-seq data processing

All ChIP-seq raw fastq data were cleaned by removing the adaptor sequence. Cutadapt (version 1.16, *http://cutadapt.readthedocs.io/en/stable/guide.html*) was used for this step with the parameters -u 10 -u -15 -U 10 -U -15 -m 30. Cleaned reads were aligned to the mouse reference genome (mm10) using BWA (version 0.7.15, *http://bio-bwa.sourceforge.net*) with the default settings. Peaks calling was finished by MACS2 (version 2.1.1, *https://github.com/taoliu/MACS*) with the parameters --nomodel --keep-dup all -p 1E-10 --broad --broad-cutoff 1E-10 --extsize 147. Replicates of ChIP-seq data were pooled for downstream analysis.

#### RNA-seq data processing

All RNA-seq raw fastq data were removed of adaptor sequence as the same way of ChIP-seq data processing. Cleaned reads were mapped to the mouse reference genome (mm10) using TopHat (version 2.1.1, *http://ccb.jhu.edu/software/tophat/index.shtml*) with the default settings. The gene expression level was calculated by Cufflinks (version 2.2.1, *http://cole-trapnell-lab.github.io/cufflinks*) and normalized by fragments per kilobase of bin per million mapped reads (FPKM).

#### Comparison between different histone markers and between replicates

For the comparison in genome-wide, the entire genome was divided into massive 2 kb windows and the enrichment of modification in each bin would be considered to calculate correlation. For the comparison in enhancers, signal of modification in each enhancer was normalized by reads of bin per million mapped reads (RPM) and such enrichment was used for calculating correlation.

#### Identification of typical and super-enhancers

Enhancers were identified by the algorithm developed by Richard A. Young, 2013. Briefly, significant distal H3K27ac peaks (peak boundary 1.5 kb or peak center 3 kb away from gene TSS) were identified, and the peaks whose distance was shorter than 12.5 kb were merged together as distal enhancers. The distal enhancers were ranked by the total signal of H3K27ac, and a plot was drawn to show the increased H3K27ac signal. Then, a tangent line with slope 1 was found of the curve and the intersection point was determined as the infection point. Enhancers above this point were defined as super-enhancers; meanwhile, enhancers below this point were defined as typical enhancers.

#### Identification of H3K4me3-enriched typical and super-enhancers

Firstly, we identified significant distal H3K4me3 peaks using the same way of defining significant distal H3K27ac peaks described previously, and nearby peaks of a distance shorter than 12.5 kb were merged together. Similar to the strategy to identify super-enhancers, H3K27ac in the regions were ranked and a plot was drawn. A tangent line with slop 1 was drawn and the intersection point was found. The enhancers above the intersection point were defined as H3K4me3 super-enhancer.

## Additional files

Additional file 1: **Figure S1.** Multiple modifications on two peptides of histone H3 and H4. **Figure S2.** The proteomic pattern of histone modifications in breast cancer model. **Figure S3.** Correlation of H3K27ac and H4K8ac distribution on chromatin. **Figure S4.** Enhancer analysis of normal tissues. **Figure S5.** H3K4me3 super enhancer in normal tissues. **Figure S6.** UCSC browser view showing the histone modification on selected genes. **Figure S7.** ChIP-PCR verification for H3K4me3 ChIP-Seqresults. **Figure S8.** Quality analysis of H3K4me3 ChIP-Seqdata with EMD 04-745. (PDF 29519 kb)

Additional file 2: **Table S1.** List of the relative abundance of all histone peptides. **Table S2.** List of the relative abundance of histone H3 and H4 peptides. **Table S3.** List of the relative abundance of single PTMs on histone H3 and H4. **Table S4.** Identified super-enhancers in tumor. **Table S5.** Identified H3K4me3 enriched super enhancers in tumor. (XLSX 189 kb)
