# Supplementary material for: The hyper-activation of transcriptional enhancers in breast cancer
Source: Clin Epigenetics. 2019 Mar 12;11:48. doi: 10.1186/s13148-019-0645-x (PMC6417266; doi:10.1186/s13148-019-0645-x)
Supplement: Supplementary file 1 — Figure S1. Multiple modifications on two peptides of histone H3 and H4. Figure S2. The proteomic pattern of histone modifications in breast cancer model. Figure S3. Correlation of H3K27ac and H4K8ac distribution on chromatin. Figure S4. Enhancer analysis of normal tissues. Figure S5. H3K4me3 super enhancer in normal tissues. Figure S6. UCSC browser view showing the histone modification on selected genes. Figure S7. ChIP-PCR verification for H3K4me3 ChIP-Seqresults. Figure S8. Quality analysis of H3K4me3 ChIP-Seqdata with EMD 04-745. (PDF 29519 kb) [file 13148_2019_645_MOESM1_ESM.pdf]

Supplemental Fig. S1

A

H3 27-40 KSAPATGGVKKPHR +2 ions

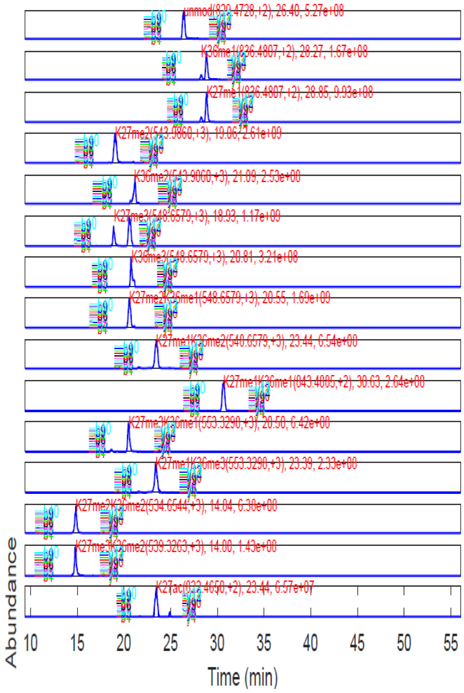

Forms	Normal			Tumor		
	rep1(%)	rep2(%)	rep3(%)	rep1(%)	rep2(%)	rep3(%)
KSAPATGGVKKPHR	4.85	3.47	2.42	3.86	4.89	3.41
KSAPATGGVK(me1)KPHR	0.80	2.38	0.47	2.28	1.54	1.86
K(me1)SAPATGGVKKPHR	12.13	8.54	8.81	6.24	8.33	5.37
K(me2)SAPATGGVKKPHR	32.08	34.03	28.88	24.62	23.65	25.06
KSAPATGGVK(me2)KPHR	3.42	2.22	0.65	1.73	2.08	2.23
K(me3)SAPATGGVKKPHR	5.87	6.99	8.44	8.45	8.32	8.82
KSAPATGGVK(me3)KPHR	7.81	9.74	2.13	2.76	2.90	1.91
K(me2)SAPATGGVK(me1)KPHR	8.44	5.45	13.99	15.72	13.09	13.88
K(me1)SAPATGGVK(me2)KPHR	5.99	3.75	3.04	6.22	6.20	6.85
K(me1)SAPATGGVK(me1)KPHR	3.16	1.83	1.54	2.92	2.56	2.64
K(me3)SAPATGGVK(me1)KPHR	2.50	4.10	6.08	4.70	4.21	4.98
K(me1)SAPATGGVK(me3)KPHR	1.50	1.49	1.21	1.87	1.87	1.88
K(me2)SAPATGGVK(me2)KPHR	10.01	12.95	17.09	14.85	16.60	17.08
K(me3)SAPATGGVK(me2)KPHR	1.43	3.06	5.23	3.73	3.71	4.02
K(ac)SAPATGGVKKPHR	0.00	0.00	0.00	0.04	0.04	0.01
Total	100	100	100	100	100	100

B

H4 4-17 GKGGKGLGKGGAKR +2 ions

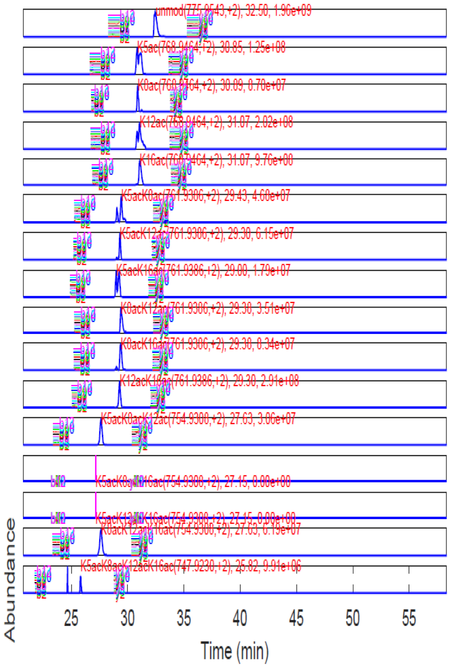

Forms	Normal			Tumor		
	rep1(%)	rep2(%)	rep3(%)	rep1(%)	rep2(%)	rep3(%)
GKGGKGLGKGGAKR	60.56	62.38	36.54	56.72	48.64	48.71
GK(ac)GGKGLGKGGAKR	3.90	3.19	6.08	5.32	4.77	5.46
GKGGK(ac)GLGKGGAKR	1.27	1.26	1.84	1.41	1.46	1.43
GKGGKGLGK(ac)GGAKR	3.97	2.90	8.28	6.64	7.95	8.08
GKGGKGLGKGGAK(ac)R	25.83	26.24	34.32	18.60	23.12	26.20
GK(ac)GGK(ac)GLGKGGAKR	0.00	0.16	0.38	1.14	1.23	0.60
GK(ac)GGKGLGK(ac)GGAKR	0.00	0.30	1.58	0.64	0.59	0.30
GK(ac)GGKGLGKGGAK(ac)R	0.93	0.59	2.06	0.56	1.12	1.33
GKGGK(ac)GLGK(ac)GGAKR	0.00	0.43	0.10	0.59	0.54	0.66
GKGGK(ac)GLGKGGAK(ac)R	1.08	0.74	1.00	1.41	2.71	1.41
GKGGKGLGK(ac)GGAK(ac)R	1.74	1.22	5.91	4.82	5.13	4.01
GK(ac)GGK(ac)GLGK(ac)GGAKR	0.12	0.11	0.55	0.60	0.75	0.48
GK(ac)GGK(ac)GLGKGGAK(ac)R	0.00	0.01	0.00	0.00	0.00	0.00
GK(ac)GGKGLGK(ac)GGAK(ac)R	0.00	0.02	0.00	0.00	0.00	0.00
GKGGK(ac)GLGK(ac)GGAK(ac)R	0.50	0.37	1.31	1.48	1.88	1.29
GK(ac)GGK(ac)GLGK(ac)GGAK(ac)R	0.10	0.09	0.07	0.07	0.12	0.04
Total	100	100	100	100	100	100

Supplemental Fig. S1. Histone peptide H3 27-40 KSAPATGGVKKPHR **(A)** and H4 4-17 GKGGKGLGKGGAKR **(B)** with multiple PTMs were analyzed by LC-MS/MS in DIA mode and quantified in relative abundance by EpiProfile. (On the left) the layouts display the extracted ion chromatography (XIC) of each peptide based on the precursor m/z (x axis -retention time, y axis-the intensity). The modification form, precursor m/z, charge state, retention time and peak intensity of each peptide are listed on top of the peak. Fragment ions (b-ion and y-ion series) are annotated on both sides of the peak. (On the right) the relative abundances of each histone peptide across all conditions and replicates are listed for comparison.

Supplemental Fig. S2

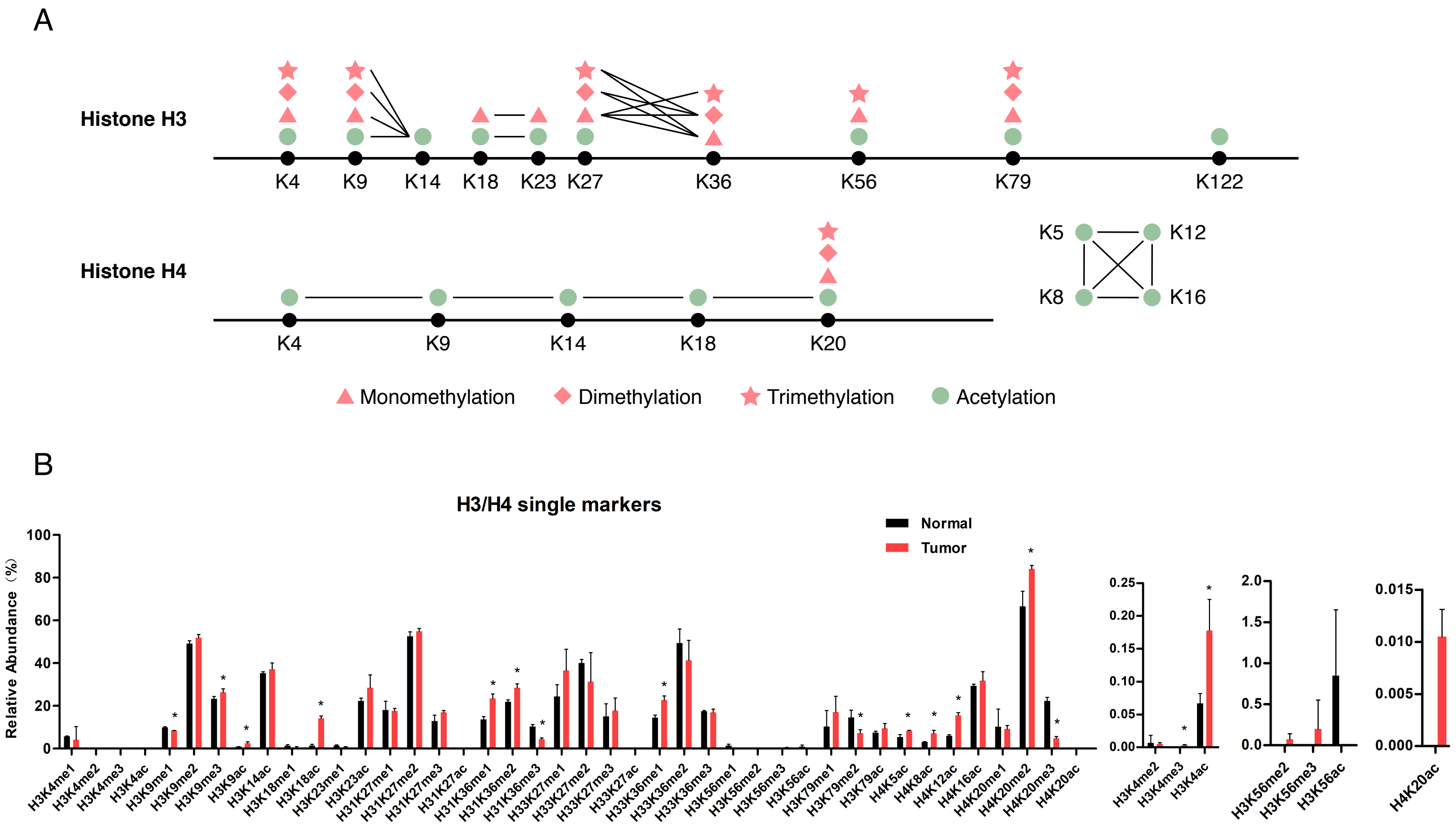

Supplemental Fig. S2. The proteomic pattern of histone modifications in breast cancer model (A) Multiple modification patterns on histone H3 and H4. Coexisting PTMs are illustrated by connecting lines. **(B)** Relative abundance of single marks on histone H3 and H4. For statistical analysis, Student's t test was used for comparison. * stands for $P < 0.05$ as compared to normal tissues.

Supplemental Fig. S3

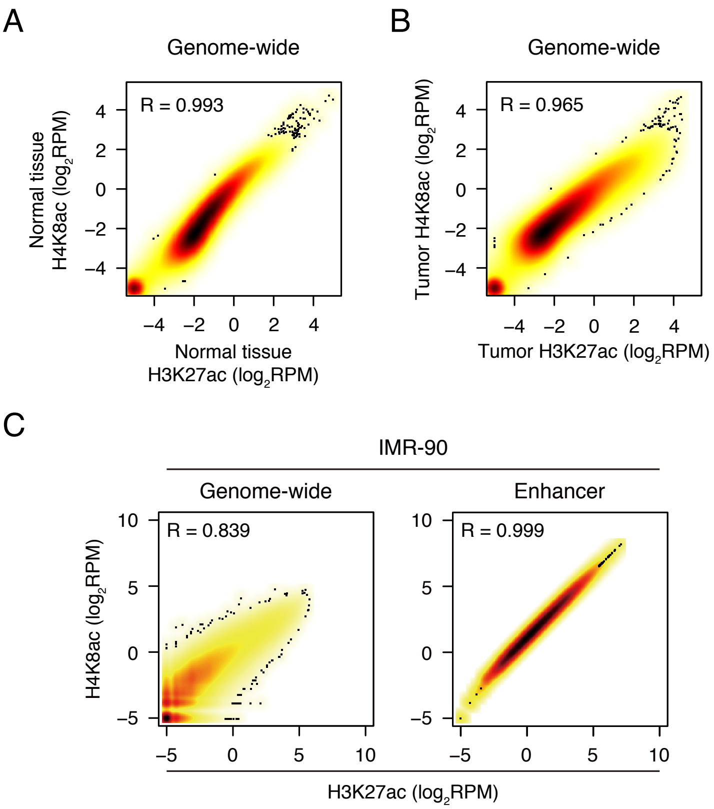

Supplemental Fig. S3 Correlation of H3K27ac and H4K8ac distribution on chromatin. (A-B) Scatter plots showing the correlation of H3K27ac and H4K8ac levels at the range of genome-wide (2-kb window) in both normal tissue and tumor. Correlation were calculated by Pearson correlation coefficient. **(C)** Scatter plots showing the correlation of H3K27ac and H4K8ac levels in genome-wide (2-kb window) (left) and enhancers (right) of IMR90 cell line. Correlation were calculated by Pearson correlation coefficient.

Supplemental Fig. S4

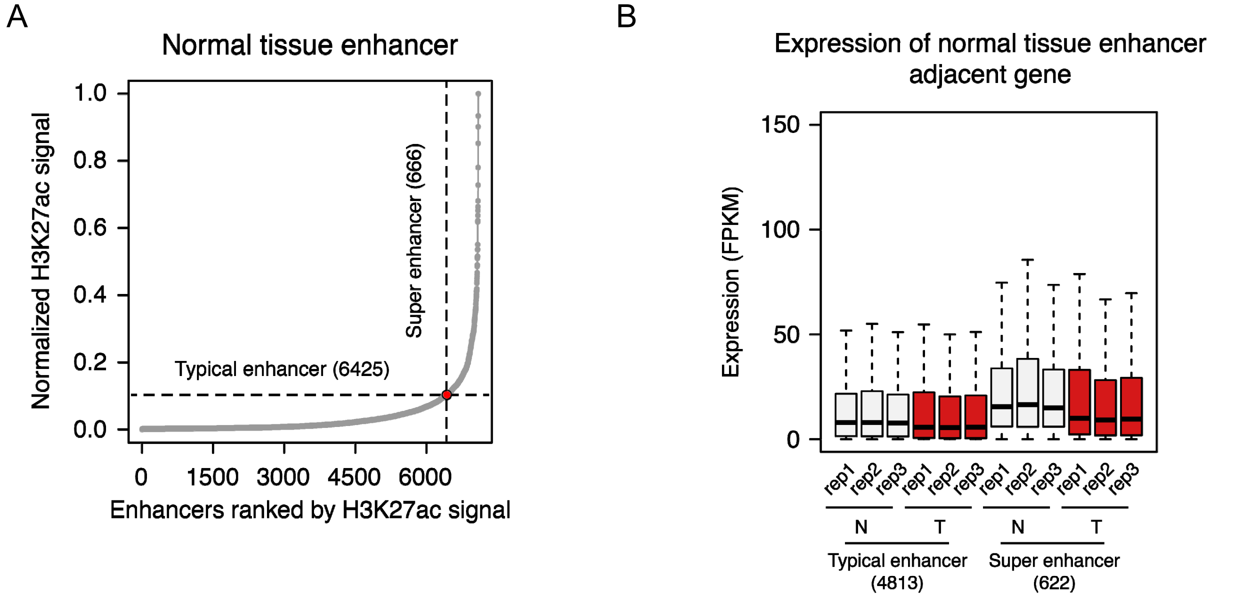

Supplemental Fig. S4. Enhancer analysis of normal tissues (A) Distribution of H3K27ac ChIP-seq signal (normalize by ratio to highest H3K27ac reads number of enhancer) across the 7,091 normal tissue enhancers. **(B)** Box plot of expression (FPKM) from normal tissue typical-enhancer-, super-enhancer-adjacent genes.

Supplemental Fig. S5

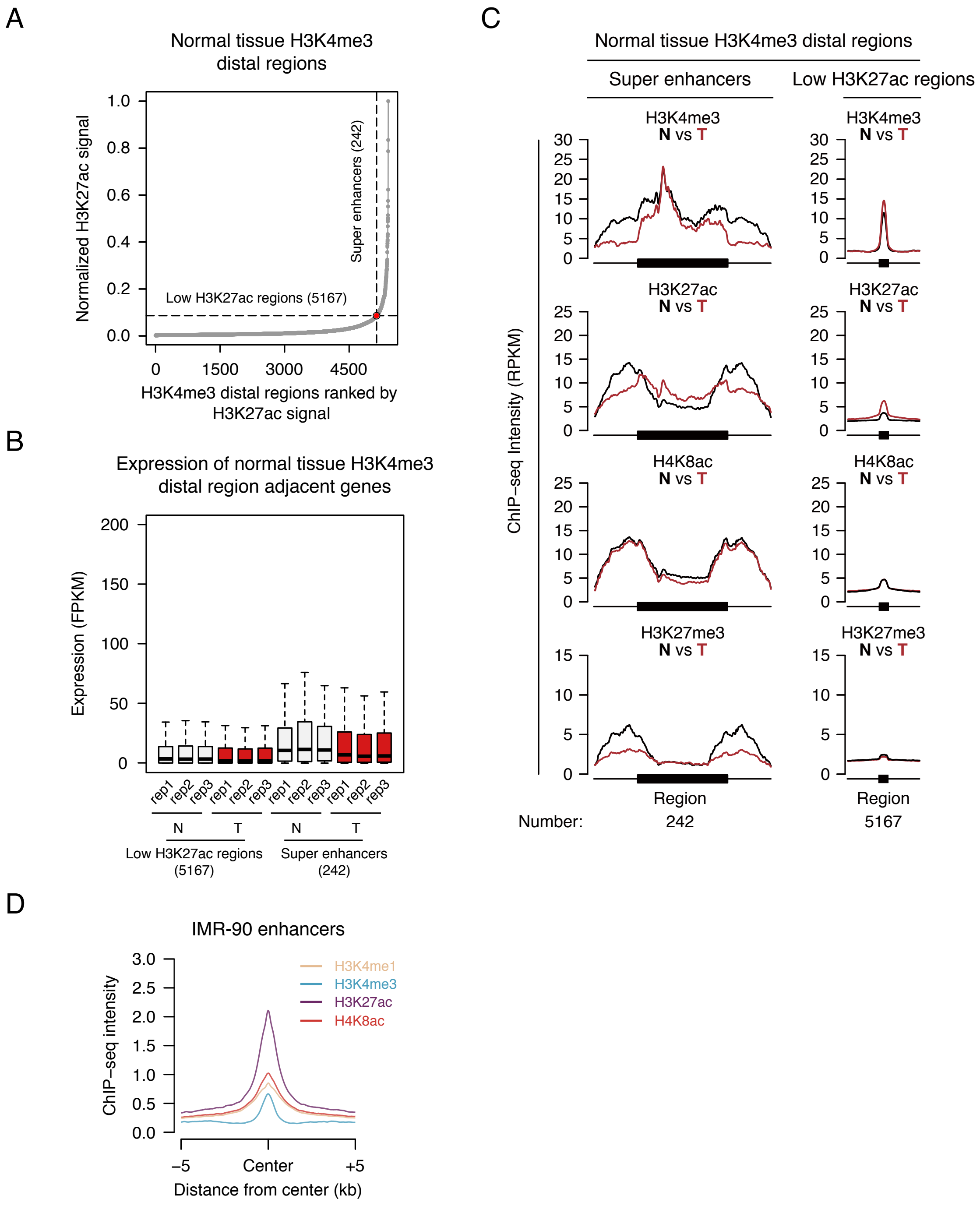

Supplemental Fig. S5. H3K4me3 super enhancer in normal tissues (A) Distribution of H3K27ac ChIP-seq signal (normalize by ratio to highest H3K27ac reads number of enhancer) across the 4,048 normal tissue 2193 H3K4me3 enriched enhancers. **(B)** Box plot of expression (FPKM) from normal tissue H3K4me3 enriched typical and super enhancer adjacent genes in both normal tissue and tumor. **(C)** Metagenes analysis of mean histone marks H3K4me3, H3K27ac, H4K8ac and H3K27me3 ChIP-seq density across normal tissue H3K4me3 enriched super and typical enhancers. Metagenes are centered on the enhancer region. The number and mean size of super and typical enhancers are shown. **(D)** Metagene analysis representation of the 4 indicated histone marks mean signals in the positions of IMR-90 cell line enhancer. The ChIP-seq data of IMR-90 4 histone marks came from ENCODE publicly available data.

Supplemental Fig. S6

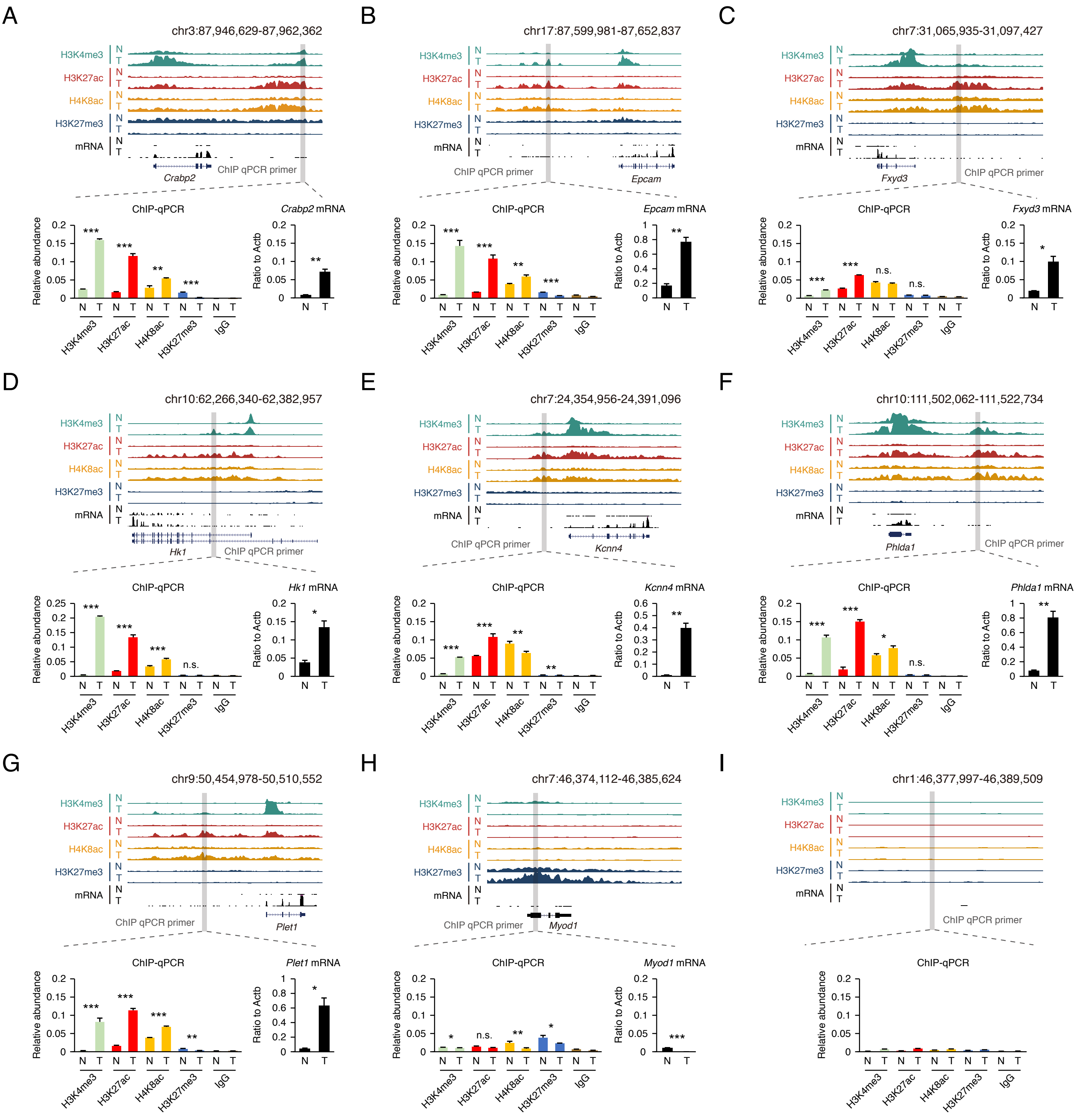

Supplemental Fig. S6. UCSC browser view on the top showing the histone modification density of H3K4me3, H3K27ac, H4K8ac, H3K27me3 and mRNA levels in gene Crabp2 (A), Epcam (B), Fxyd3 (C), Hk1 (D), Kcnn4 (E), Phlda1 (F), Plet1 (G), Myod1 (H) locus and a negative control position (I). Bar plot on bottom left in S4. A-H or bottom in S4. I showing the results of ChIP-qPCR in positions highlighted by grey bar. Bar plot on bottom right in S4. A-H illustrating the mRNA level of gene labeled in the top UCSC browser view by q-PCR. q-PCR data are represented as mean ± SD from three technical replicates in ChIP and two in mRNA. * $p < 0.05$; ** $p < 0.01$; *** $p < 0.001$; t test.

Supplemental Fig. S7

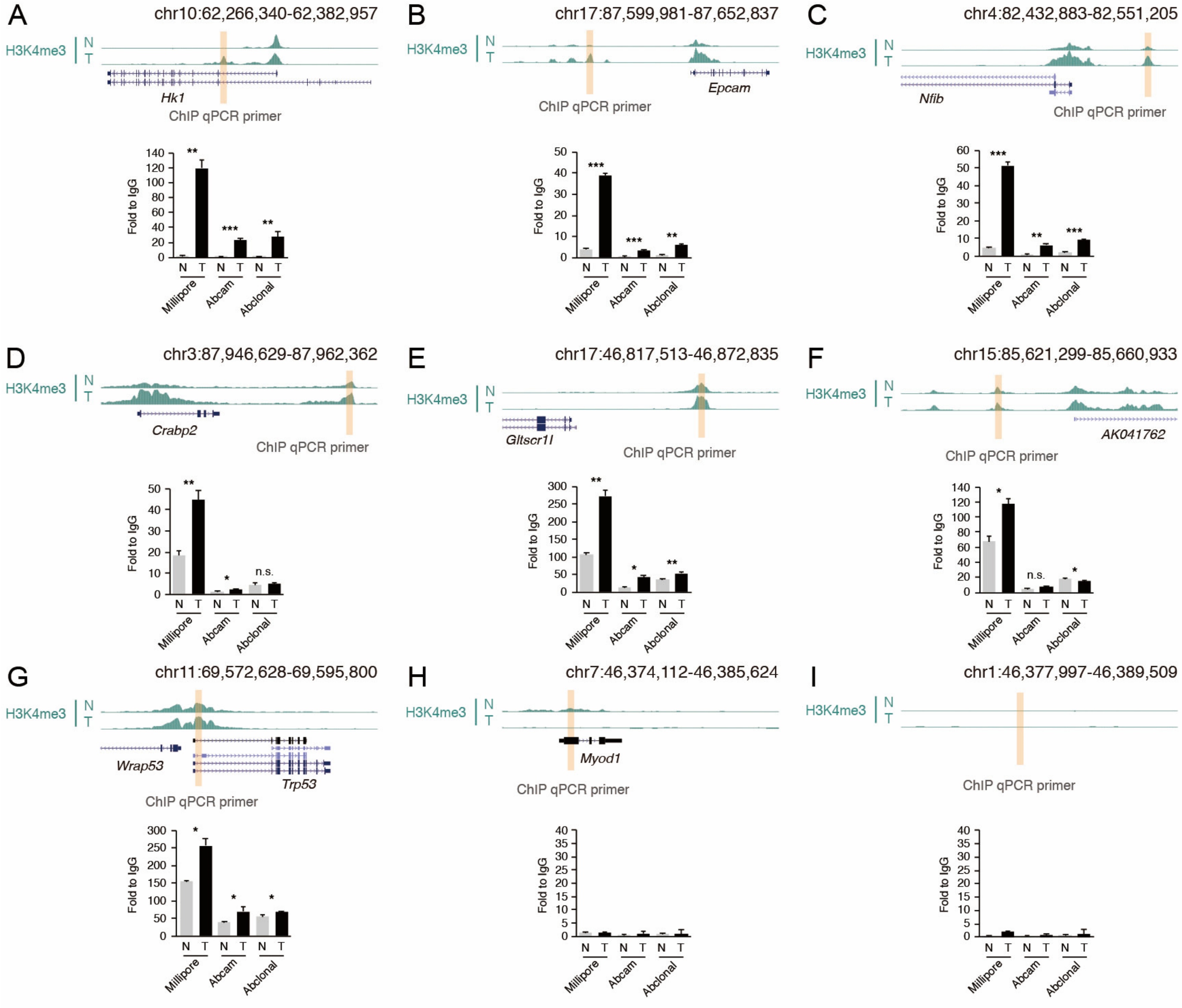

Supplemental Fig. S7 ChIP-PCR verification for H3K4me3 ChIP-Seq results. ChIP assays of H3K4me3 from three vendors (EMD 04-745, Abcam ab213224 and Abclonal A2357) were performed with fresh-made control and tumor tissues. EMD 04-745 was the antibody used in ChIP-Seq. Six enhancers with enhanced H3K4me3 in tumors **(A-F)**, one promoter **(G)** and two negative sites **(H & I)** were selected. H3K4me3 (Millipore) ChIP-Seq signals were shown on the top of each panel. The brown blocks label the designed PCR products.

Supplemental Fig. S8

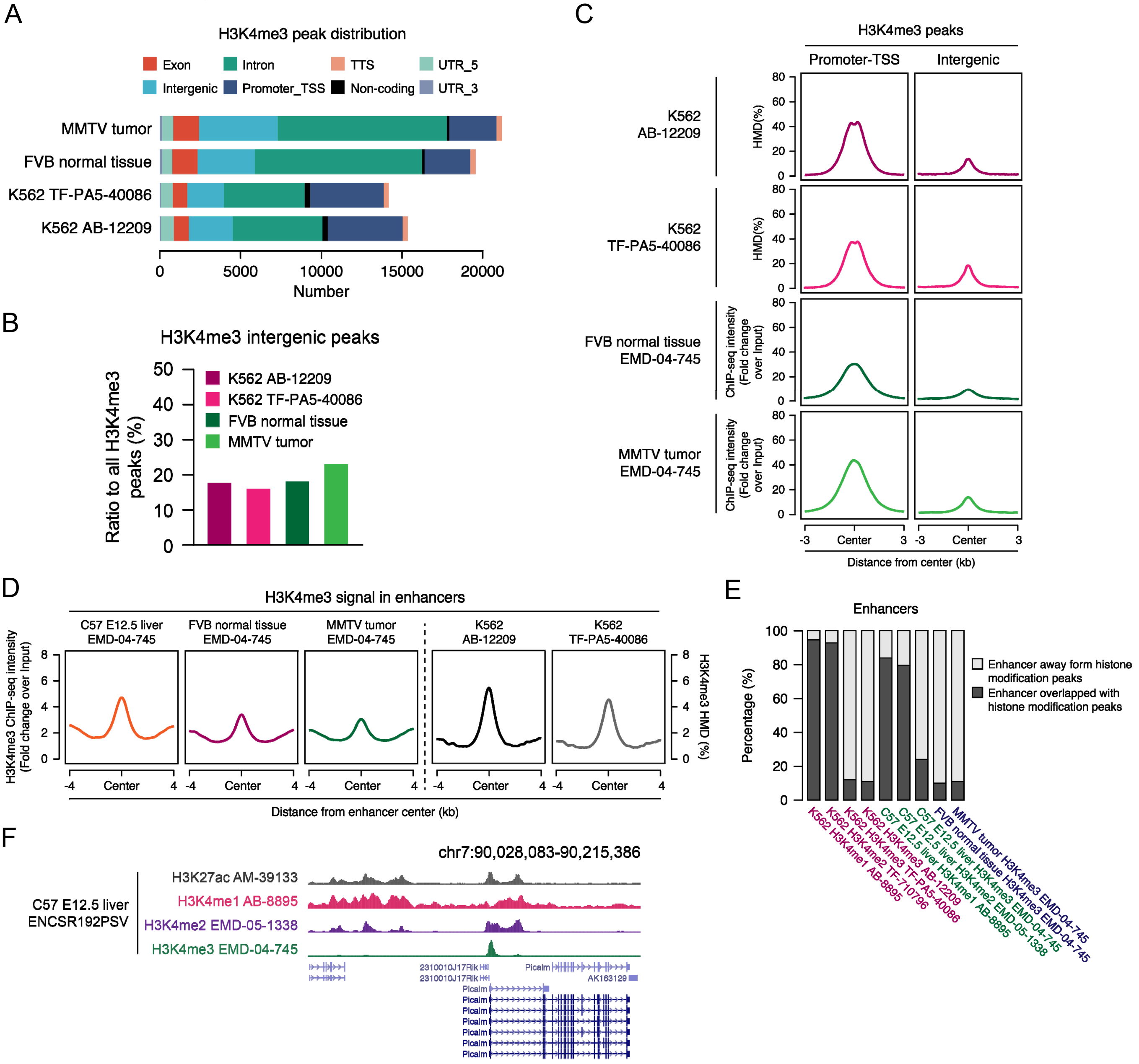

Supplemental Fig. S8 Quality analysis of H3K4me3 ChIP-Seq data with EMD 04-745. (A) The distribution of H3K4me3 peaks of 4 samples in different genome elements, calculated by HOMER module *annotatePeaks.pl*. . Promoter-TSS, "-1kb to +100bp" of transcription start sites. TTS, "-100bp to +1Kb" of transcription termination sites. **(B)** The ratio of H3K4me3 intergenic peaks to all H3K4me3 peaks according to Fig. A. **(C)** The metagene of H3K4me3 signal in promoter-TSS and intergenic regions. Top 2 samples H3K4me3 signal were normalized using histone modification density (HMD) with the following formula: HMD (%) = 100% * (IPlocus / INlocus) / (IPbarcode / INbarcode). Bottom 2 samples signal were normalized with fold change over Input, with the following formula: ChIP-seq intensity = IPlocus / INlocus. **(D)** The metagene of H3K4me3 signal on enhancers identified with H3K27ac ChIP-seq data. The left three samples were normalized with fold change over Input, and the right two samples with HMD **(E)** The proportion of enhancers which overlapped with indicated histone modifications. **(F)** UCSC genome browser view showing the signal of H3K27ac and H3K4me1/2/3 in Picalm gene locus. These data were public and performed by ENCODE project (ENCSR192PSV).

RESOURCE For Supplemental Fig. S8

Sample	Species	Histone marker	Antibody	Source
K562	Human	H3K4me1	Abcam ab8895	GEO: GSE103543 - GSM2773392
K562	Human	H3K4me2	Thermo Fisher 710796	GEO: GSE103543 - GSM2773400
K562	Human	H3K4me3	Thermo Fisher PA5-40086	GEO: GSE103543 - GSM2773406
K562	Human	H3K4me3	Abcam ab12209	GEO: GSE103543 - GSM2773401
C57BL/6 E12.5 liver	Mouse	H3K4me1	Abcam ab8895	ENCODE: ENCSR770OXU
C57BL/6 E12.5 liver	Mouse	H3K4me2	EMD Millipore 05-1338	ENCODE: ENCSR110MSZ
C57BL/6 E12.5 liver	Mouse	H3K4me3	EMD Millipore 04-745	ENCODE: ENCSR471SJG
FVB mouse mammary gland	Mouse	H3K4me3	EMD Millipore 04-745	This paper
MMTV mammary tumor	Mouse	H3K4me3	EMD Millipore 04-745	This paper
